# A Hyperthermoactive-Cas9 Editing Tool Reveals the Role of a Unique Arsenite Methyltransferase in the Arsenic Resistance System of Thermus thermophilus HB27

**DOI:** 10.1128/mBio.02813-21

**Published:** 2021-12-07

**Authors:** Giovanni Gallo, Ioannis Mougiakos, Mauricio Bianco, Miriam Carbonaro, Andrea Carpentieri, Anna Illiano, Pietro Pucci, Simonetta Bartolucci, John van der Oost, Gabriella Fiorentino

**Affiliations:** a Department of Biology, University of Naples Federico II, Naples, Italy; b Laboratory of Microbiology, Wageningen University & Research, Wageningen, The Netherlands; c Department of Chemical Sciences, University of Naples Federico II, Naples, Italy; d Consiglio Nazionale delle Ricerche CNR, Institute of Polymers, Composites and Biomaterials (IPCB), Pozzuoli, Naples, Italy; e Helmholtz Institute for RNA-based Infection Research (HIRI), Helmholtz-Centre for Infection Research (HZI), Würzburg, Germany; f CEINGE-Biotecnologie avanzate, Naples, Italy; g Task Force on Microbiome Studies, University of Naples Federico II, Naples, Italy; Friedrich-Schiller-Universitat; Karlsruhe Institute of Technology (KIT)

**Keywords:** arsenic resistance, thermoresistance, extremophiles, *Thermus thermophilus*, CRISPR-Cas9 genome editing, genetic tool, bioreporter

## Abstract

Arsenic detoxification systems can be found in a wide range of organisms, from bacteria to humans. In a previous study, we discovered an arsenic-responsive transcriptional regulator in the thermophilic bacterium Thermus thermophilus HB27 (*Tt*SmtB). Here, we characterize the arsenic resistance system of T. thermophilus in more detail. We employed *Tt*SmtB-based pulldown assays with protein extracts from cultures treated with arsenate and arsenite to obtain an *S*-adenosyl-l-methionine (SAM)-dependent arsenite methyltransferase (*Tt*ArsM). *In vivo* and *in vitro* analyses were performed to shed light on this new component of the arsenic resistance network and its peculiar catalytic mechanism. Heterologous expression of *TtarsM* in Escherichia coli resulted in arsenite detoxification at mesophilic temperatures. Although *Tt*ArsM does not contain a canonical arsenite binding site, the purified protein does catalyze SAM-dependent arsenite methylation with formation of monomethylarsenites (MMAs) and dimethylarsenites (DMAs). In addition, *in vitro* analyses confirmed the unique interaction between *Tt*ArsM and *Tt*SmtB. Next, a highly efficient ThermoCas9-based genome-editing tool was developed to delete the *Tt*ArsM-encoding gene on the T. thermophilus genome and to confirm its involvement in the arsenite detoxification system. Finally, the *TtarsX* efflux pump gene in the T. thermophilus Δ*TtarsM* genome was substituted by a gene encoding a stabilized yellow fluorescent protein (sYFP) to create a sensitive genome-based bioreporter system for the detection of arsenic ions.

## INTRODUCTION

Arsenic is the most abundant environmental toxic element which enters the biosphere mainly from geochemical and (to a lesser extent) anthropogenic sources such as herbicides, growth promoters for livestock, and industrial activities (1). Arsenic has two relevant oxidation states, trivalent arsenite [As(III)] and pentavalent arsenate [As(V)]. Methylated arsenicals include mono- (MA), di- (DMA), and tri- (TMA) methylated forms. In general, trivalent states are more toxic than pentavalent ones, and TMAs are more toxic than inorganic arsenite. Although arsenic is not beneficial for life, it can enter cells through transporters such as aquaglyceroporins. Hence, arsenic detoxification systems can be found in a wide range of organisms, from bacteria to humans. Arsenic resistance genes (*ars*) include genes encoding efflux transporters, redox enzymes, methyltransferases, transcriptional repressors, and biosynthetic pathways for arsenosugars and arsenolipids (2, 3). The identification and characterization of these pathways have attracted the attention of fundamental, evolutionary, and biotechnological research (4–6).

Microorganisms have been exposed to arsenic since the origin of life and consequently have evolved arsenic resistance systems, encoded by genes generally clustered in operons (7). The organization and number of the operons of arsenic resistance genes are highly variable between different species (8, 9), reflecting differences in the level of arsenic resistance. The key players of arsenic detoxification are (i) arsenate reductases (ArsC) that reduce intracellular arsenate to arsenite (10), (ii) efflux permeases responsible for arsenite transport outside the cell (11), and (iii) transcriptional repressors that are generally metalloregulatory proteins of the ArsR/SmtB family (12). In addition, arsenite can be methylated by arsenite *S*-adenosylmethionine methyltransferases (ArsM) into MMAs, DMAs, and TMAs (13), after which they can passively leave the cell or be extruded by methylarsenite-specific efflux permease (ArsP) (14).

Based on recent molecular clock analyses, it has been concluded that arsenite efflux and arsenite methylation represented the core of microbial arsenic resistance systems before the rise of atmospheric oxygen (15). In such primordial anoxic environments, methyl-arsenicals could also function as antibiotics against competitor microbes. After the rise of atmospheric oxygen, the ArsM enzymes did become primary components of the arsenic detoxification machinery; nevertheless, in some microorganisms, they maintained their antibiotic activity (16, 17).

Hydrothermal hot springs, which can be considered environments with conditions similar to niches of primordial Earth, may contain large amounts of arsenic. In these cases, these hot springs are niches for arsenic-tolerant microorganisms, which play a critical role in the global arsenic biogeochemical cycle (18). Although the resistance mechanisms to inorganic arsenic have been studied in many microorganisms (19), the contribution of organo-arsenical biotransformation in extreme environments is still at a stage of infancy. In this regard, only two algal thermoactive ArsM enzymes have been characterized to date (20).

The thermophilic bacterium Thermus thermophilus HB27, originally isolated from a volcanic hot spring in Japan (21), has an unusual genetic organization of its machinery to cope with arsenic toxicity. The currently identified arsenic resistance genes are randomly scattered in its genome (22), complicating the identification of all the genes involved. In previous studies, we elucidated some components of the arsenic resistance system of T. thermophilus HB27. We identified and characterized *Tt*SmtB, the metalloregulatory transcriptional repressor that is responsible for the regulation of the arsenic detoxification system. *Tt*SmtB recognizes and firmly binds to operator sequences in the promoter regions of the arsenite efflux gene (*TtarsX*) (23) and the arsenate reductase gene (*Tarec*) (24, 25), efficiently repressing their transcription in the absence of arsenic ions. *Tt*SmtB and *Tt*ArsX are also involved in cadmium sensing and export, respectively (23). In two T. thermophilus HB27 deletion mutant strains (Δ*TtarsX* and Δ*TtsmtB*), tolerance to arsenate, arsenite, and cadmium was significantly reduced compared to the wild-type strain. Although these analyses confirmed the involvement of *Tt*ArsX and *Tt*SmtB in the promiscuous resistance mechanism, the mutant strains could still grow at concentrations of arsenic up to 3 mM (22, 23). Notably, the genome of T. thermophilus HB27 is not predicted to express arsenite methyltransferases or arsenite oxidases, suggesting the existence of unidentified component(s) of the T. thermophilus HB27 arsenic resistance system that cannot be predicted by *in silico* approaches, highlighting the need to employ an alternative experimental identification method.

Since members of the ArsR/SmtB family are a group of homodimeric proteins with a common HTH-winged helix DNA binding domain and heterogeneous metal binding domain architectures and interaction modes (26), we hypothesized that *Tt*SmtB could even form protein interactions with unknown, functionally related protein partners.

In this study, using an integrated proteomic, biochemical, and genetic approach, we provide a gain of insight into the arsenic resistance system of T. thermophilus HB27. We report the discovery of the first T. thermophilus HB27 arsenite methyltransferase, *Tt*ArsM. Moreover, we describe the development of a highly efficient, markerless Cas9-based genome-editing tool at temperatures up to 65°C. Using this ThermoCas9 system, we demonstrated the *in vivo* involvement of *Tt*ArsM in arsenite detoxification. The newly developed genome editing tool was further validated by constructing a very sensitive whole-cell bioreporter system in which the *TtarsX* efflux transporter gene was substituted by a gene encoding a thermo-adapted superfolder yellow fluorescent protein (*syfp*) (27).

## RESULTS

### Exploring the protein-protein interactions of *Tt*SmtB.

A combined comparative and functional proteomic approach was employed to identify putative *Tt*SmtB-interacting proteins with a role in arsenite metabolism/detoxification. Purified recombinant His-tagged *Tt*SmtB was bound to a Ni^2+^-nitrilotriacetic acid (NTA) resin for protein pulldown assays using T. thermophilus HB27 cell extracts (CFE) from cultures exposed either to arsenite or arsenate or untreated CFE cultures used as control. SDS-PAGE separation of the pulled-down proteins eluted with 0.5 M imidazole, followed by liquid chromatography-electrospray ionization-tandem mass spectrometry (LC-ESI-MS/MS) (28), and comparative analysis of the acquired data resulted in the identification of 51 cytosolic proteins that interact with *Tt*SmtB (Table S1 in the supplemental material). Only five of these proteins are simultaneously present in CFE from cultures exposed to arsenite and arsenate, but not in control CFE from the nonexposed cultures. Among these proteins, TTC0109 (GenPept accession no. AAS80457; UniProt code Q72LF0) was predicted to be involved in the arsenic detoxification system (based on homology to annotated ArsR family transcriptional regulators) and to contain a C-terminal *S*-adenosyl-l-methionine (SAM)-dependent methyltransferase domain (based on homology to annotated methyltransferase domain-containing proteins), suggesting a role in arsenic methylation. To date, there are no annotated arsenite SAM-dependent methyltransferases in the genome of T. thermophilus HB27 or the genomes of other thermophilic bacteria. Hence, we selected TTC0109 for further investigation as a potential novel arsenite methyltransferase.

10.1128/mBio.02813-21.1 TABLE S1 List of *Tt*SmtB cytosolic interactors. Download Table S1, DOCX file, 0.02 MB. Copyright © 2021 Gallo et al. 2021 Gallo et al. https://creativecommons.org/licenses/by/4.0/ This content is distributed under the terms of the Creative Commons Attribution 4.0 International license.

### Bioinformatics analysis of TTC0109.

**FIG 1:**
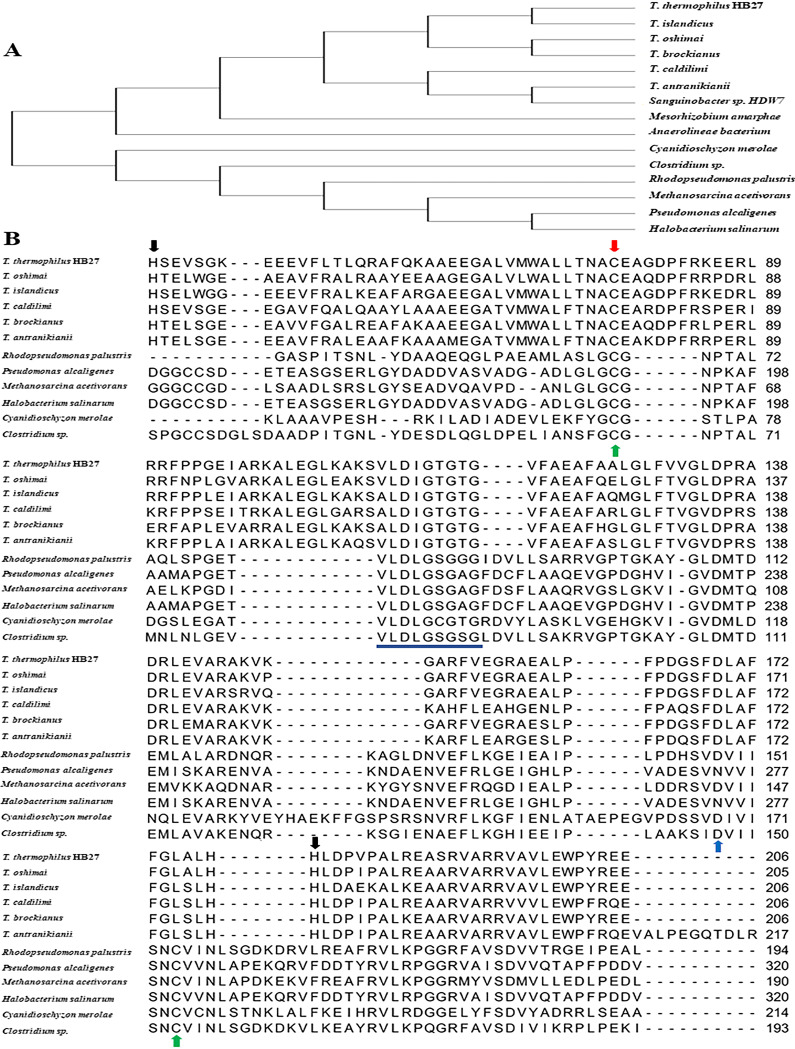
(A) Phylogenetic tree of archaeal and bacterial arsenite methyltransferases, SAM-dependent methyltransferases, and methyltransferase domain-containing proteins. The aminoacidic sequences used for the construction of the phylogenetic tree are *Tt*ArsM from T. thermophilus HB27; SAM-dependent methyltransferase from five members of the *Thermus* genus (T. islandicus, T. caldilimi, T. antranikianii, T. oshimai, and T. brockianus), Anaerolineae bacterium, and Mesorhizobium amorphae; arsenite methyltransferase from *Clostridium* sp. strain BMX (55), Methanosarcina acetivorans, Rhodopseudomonas palustris (13), Pseudomonas alcaligenes (38), Halobacterium salinarum (56), and Cyanidioschyzon merolae (45); and a methyltransferase domain-containing protein from *Sanguinobacter* sp. (B) Multiple-sequence alignment of hypothetical and functionally characterized arsenite methyltransferases (ArsM) with T. thermophilus HB27 *Tt*ArsM. The partial alignment includes 5 members of the *Thermus* genus (T. islandicus, T. caldilimi, T. antranikianii, T. oshimai, and T. brockianus) (98% identity to *Tt*ArsM, sequence aligned from amino acids 40 to 89), *Clostridium* sp. BMX (55) (28% identity to *Tt*ArsM, from 22 to 193), R. palustris (13) (32% identity to *Tt*ArsM, from 23 to 168), M. acetivorans (39) (29% identity to *Tt*ArsM, from 19 to 190), P. alcaligenes (38) (31% identity to *Tt*ArsM, from 149 to 320), Cyanidioschyzon merolae (45) (27.7% identity to *Tt*ArsM, from 29 to 214), and H. salinarum (56) (25% identity to *Tt*ArsM, from 58 to 294). Red arrow indicates the catalytic cysteine conserved in characterized ArsM and *Tt*ArsM; green arrows indicate two catalytic cysteines conserved in characterized ArsM, but not in *Tt*ArsM; blue arrow indicates the conserved aspartic acid; and the SAM binding domain, which is part of the typical Rossman fold, is underlined in blue. The two histidines of *Tt*ArsM predicted to interact with arsenite are indicated by black arrows.

BLASTP analysis of TTC0109 translated sequence with sequenced microbial genomes and evolutionary analysis conducted with MEGA X demonstrated that TTC0109 is highly conserved among the members of the *Thermus* genus (Fig. 1A). Moreover, multiple-sequence alignment of TTC0109 with characterized prokaryotic arsenite methyltransferases (Fig. 1B) showed that all the aligned proteins contain a typical Rossman fold (29). This fold contains a GxGxG motif in a loop region, which presumably interacts with the carboxypropyl moiety of SAM, and a highly conserved aspartic acid residue at the end of the β2 strand, which forms hydrogen bonds with the ribose hydroxyls of the cofactor (30). In the case of TTC0109, the predicted GxGxG motif is composed of G114, T115, G116, T117, and G118 residues, and the conserved aspartic residue is D135 (29, 30).

On the other hand, the alignment shows that TTC0109 greatly differs from characterized ArsM proteins in the remaining sequence (Fig. 1B); nonetheless, if TTC0109 is an arsenite methyltransferase, it would be evolutionarily distant from other archaeal and bacterial arsenite methyltransferases, as shown in the phylogenetic tree (Fig. 1A), and therefore, it could belong to a new type of arsenite methyltransferases. Additionally, all the known arsenite methyltransferases, including those in the alignment, possess at least two, usually three, cysteines that are responsible for the binding of arsenite and its subsequent methylation (16, 31). TTC0109 contains a single cysteine residue at position 77, which is perfectly conserved in all the sequences analyzed (Fig. 1B); hence, TTC0109 could be an arsenite methyltransferase with a distinct reaction mechanism.

Reasoning that the TTC0109 structure could provide more information regarding the function of TTC0109, we generated a structural model of the protein and performed molecular docking with arsenite and SAM (Fig. S1). The obtained model predicts that TTC0109 forms homodimers via its N-terminal moiety; molecular docking highlighted that arsenite could be coordinated by two histidines, H40 and H179, while C77 interacts with the methyl group of SAM (Fig. S1A).

10.1128/mBio.02813-21.6 FIG S1 TTC0109 three-dimensional model (wild type and mutants) and docking with arsenite and SAM (the arsenic atom is the purple sphere, the oxygen atoms are the red spheres, and SAM is orange). The H40 and H179 residues coordinating arsenite are colored blue. The C77 residue is colored black. (B) TTC0109 C77S; (C) TTC0109 H40A; (D) TTC0109 H179A. Blue indicates the mutated position. Download FIG S1, TIF file, 2.4 MB. Copyright © 2021 Gallo et al. 2021 Gallo et al. https://creativecommons.org/licenses/by/4.0/ This content is distributed under the terms of the Creative Commons Attribution 4.0 International license.

Although H40 and H179 residues are not conserved in characterized ArsM proteins, they are maintained at an identical position in the translated genomes of all *Thermus* species (five of them are shown in Fig. 1B); moreover, H40 is encompassed in a sequence motif (34-YRVFPTHSE-42) that shares 45% identity (underlined) with a sequence motif (101-YRLADRHVE-109) at the C terminus of the *Tt*SmtB metal binding site (32), strengthening the hypothesis that H40 could be involved in arsenite binding and suggesting an evolutionary connection between the two proteins.

We proceeded with the generation of structural models of mutant proteins in which the amino acids H40 or H179 were replaced with alanine residues producing TTC0109 H40A and TTC0109 H179A and C77 replaced with a serine residue generating TTC0109 C77S (Fig. S1B to D). The predicted models showed that the substitution of either H40 or H179 with an alanine residue altered the three-dimensional structure of TTC0109, whereas the effect of the cysteine-to-serine substitution had only a minimal effect (Fig. S1C and D), supporting the hypothesis that this residue could have a functional role.

### TTC0109 is a novel arsenite methyltransferase.

**FIG 2:**
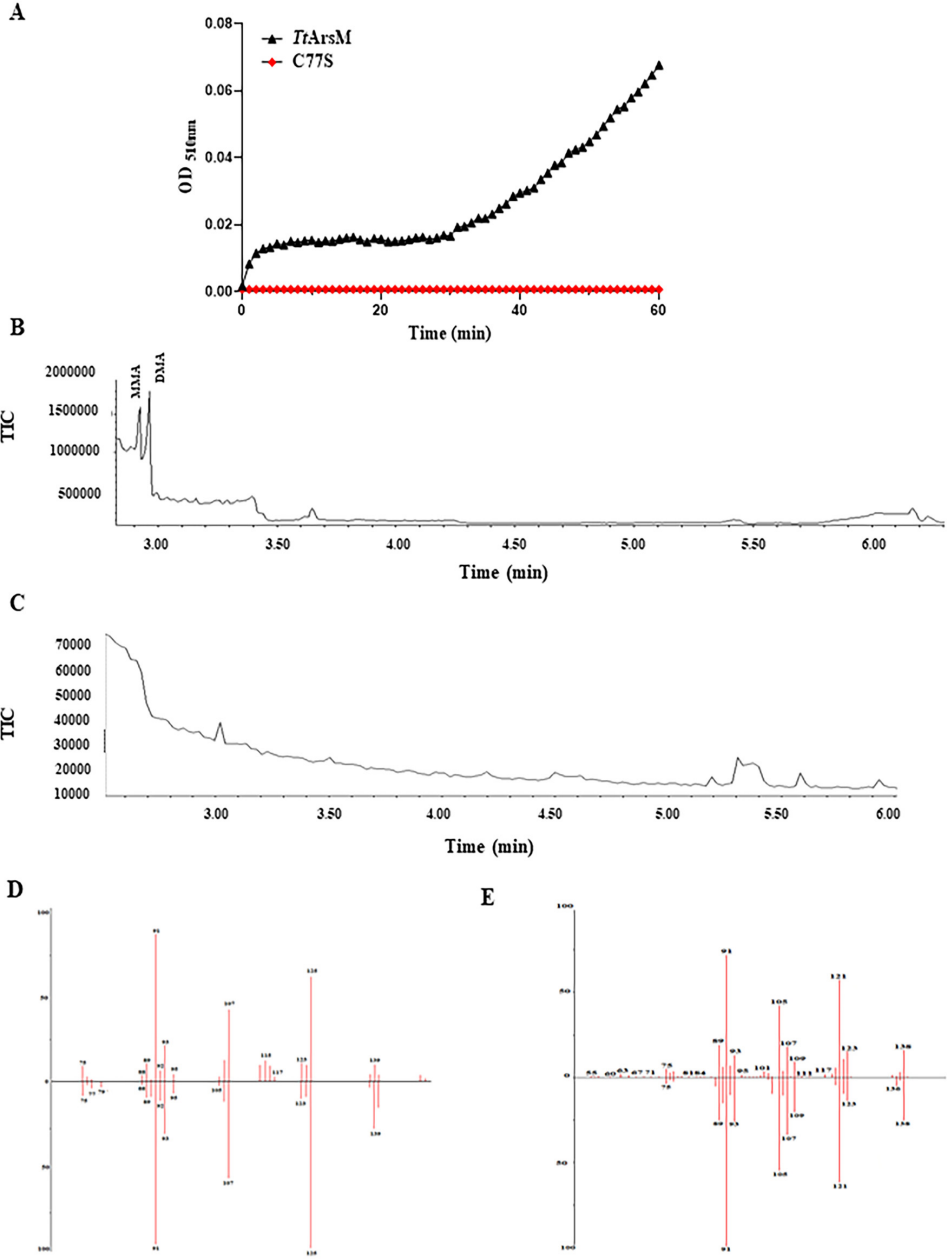
*In vitro* assessment of methyltransferase activity. (A) Arsenite methylation assays. The enzyme-coupled colorimetric assay was carried out in continuous rotation in the presence of 3.1 μM recombinant *Tt*ArsM (black curve) or *Tt*ArsM C77S (red curve), 800 μM SAM, and 200 μM As(III) at 50°C. The absorbance of the reaction mixture was recorded every minute for a total of 1 h. The graph represents the average of three independent experiments, each performed in triplicate. (B) Products of As(III) methylation by purified *Tt*ArsM. Arsenic species were analyzed by GC-MS. Each assay contained 10 μM *Tt*ArsM, 250 μM As(III), 6 mM GSH, and 1 mM SAM, incubated at 65°C for 24 h. (C) Negative control. Both chromatograms were recorded between 2 and 6 min. (D and E) Butterfly plots. The plots show the correspondence between experimental and theoretical fragmentation spectra for MMA and DMA, respectively. The plots confirmed the arsenite methyltransferase activity of *Tt*ArsM.

A recombinant His-tagged version of TTC0109 was produced and purified to homogeneity from Escherichia coli BL21-CodonPlus (DE3)-RIL cells transformed with pET30b(+)*/TtarsM* (predicted mass, 29.1 kDa) (Fig. S2A). Gel filtration chromatography analysis agreed with the *in silico*-predicted dimeric configuration of the protein, showing that the homodimer has a mass of approximately 64.5 kDa (Fig. S2B). To determine whether TTC0109 had arsenite methyltransferase activity, a coupled spectrophotometric enzymatic assay based on the formation of *S*-adenosylhomocysteine (SAH) from SAM after transfer of methyl group(s) on the substrate was employed (33, 34). In this case, the acceptor of methyl groups was As(III). SAH is degraded by SAH nucleosidase into *S*-ribosylhomocysteine and adenine; adenine deaminase acts on adenine producing hypoxanthine, which is converted into urate and hydrogen peroxide (H_2_O_2_) by xanthine oxidase. The rate of production of H_2_O_2_ is measured by an increase in absorbance at 510 nm with the help of the colorimetric reagent 3,5-dichloro-2-hydroxybenzensulfonic acid (DHBS). Then, arsenite methyltransferase activity was assayed following the increase in absorbance at 510 nm (35). Preliminary assays were set up to assess the thermal stability of the different components, and, consequently, the optimal assay temperature; afterward, the saturating concentrations of SAM and arsenite were determined. Therefore, the optimal assay conditions resulted as follows: 50°C, 200 μM arsenite, 800 μM SAM, and 3.1 μM TTC0109. Under these conditions, the specific arsenite methyltransferase activity of TTC0109 was 4.5 mU/mg (Fig. 2A). For this reason, here, the TTC0109 protein will be denoted as *Tt*ArsM.

10.1128/mBio.02813-21.7 FIG S2 Purification of recombinant *Tt*ArsM. (A) SDS-PAGE analysis. Lane M, protein marker; lane NI, protein extract from noninduced cells; lane I, protein extract from induced cells; lane P, pure protein after His-Trap chromatography. (B) Size exclusion chromatogram of *Tt*ArsM; the calibration curve is in the box. The chromatogram shows a peak corresponding to the *Tt*ArsM dimeric form. (C) Histograms represented the average peak areas for MMAs and DMAs in the *Tt*ArsM enzymatic reaction sample. These peak area values correspond to a concentration lower than the lower limit of quantification (LOQ) assessed to 1 mg/liter by using standard molecules. The coefficient of variation (CV%) values obtained were lower than 15%. Download FIG S2, TIF file, 0.4 MB. Copyright © 2021 Gallo et al. 2021 Gallo et al. https://creativecommons.org/licenses/by/4.0/ This content is distributed under the terms of the Creative Commons Attribution 4.0 International license.

In order to determine which products are formed upon As(III) methylation by *Tt*ArsM, we incubated 10 μM *Tt*ArsM with As(III), GSH, and SAM at 65°C for 24 h; the mixture was then solubilized and analyzed by gas chromatography-mass spectrometry (GC-MS) (36). Two sharp peaks, at 2.92 min and 2.96 min, were present in the chromatogram of the product of the *Tt*ArsM reaction (Fig. 2B) but not in the chromatogram of the product of the control reaction (Fig. 2C). These peaks were tentatively attributed to MMAs and DMAs, respectively. Unambiguous identification of each compound was accomplished by comparison of their retention times and fragmentation spectra with those of MMA and DMA standards. Butterfly plots showing the strong correspondence of experimental versus theoretical fragmentation spectra for MMAs and DMAs are depicted in Fig. 2D and E, respectively. The assignment was further confirmed by matching the experimental fragmentation spectra with those publicly available in the NIST 05 mass spectral library. According to NIST guidelines, score values higher than 700 indicate an effective identification; the scores determined for MMA and DMA were 790 and 820, respectively (37). MMAs and DMAs were manually integrated, and the results are summarized in Fig. S2C.

The results of *in vitro* assays using purified *Tt*ArsM protein confirmed the ability of the protein to methylate As(III)-producing mono- and dimethylated arsenic, the latter being the primary product. The oxidation state of the products could not be determined because the reactions were terminated with H_2_O_2_, which oxidized all arsenicals to pentavalent states.

Since the *in silico* predictions of *Tt*ArsM led to the hypothesis that C77, H40, and H179 were catalytic amino acids, three mutated versions of *TtarsM*, namely, *TtarsM* C77S, *TtarsM* H40A, and *TtarsM* H179A, were constructed and expressed in E. coli BL21-CodonPlus(DE3)-RIL cells, and the corresponding *Tt*ArsM mutants were purified (Fig. S3). Although the expression levels of the three mutant proteins are comparable (Fig. S3), it was not possible to perform *in vitro* characterization of purified *Tt*ArsM H40A and *Tt*ArsM H179A, which precipitated in solution after purification; this phenomenon is probably due to protein instability, thus indicating the importance of these amino acids for *Tt*ArsM structure. On the other hand, the purification of soluble *Tt*ArsM C77S protein was possible, albeit with a lower yield than the wild-type *Tt*ArsM. Nonetheless, using the previously mentioned coupled assay, this mutant enzyme did not show any *in vitro* arsenite methyltransferase activity, confirming the C77 residue plays a role in *Tt*ArsM activity (Fig. 2A). These *in vitro* results demonstrated that *Tt*ArsM has arsenite methyltransferase activity, and its distinct active site suggests a novel reaction mechanism compared to other characterized arsenite methyltransferases (13, 38, 39).

10.1128/mBio.02813-21.8 FIG S3 Purification of recombinant *Tt*ArsM mutants. SDS analysis. Lane M, protein marker; lane NI, protein extract from noninduced cells; lane I, protein extract from induced cells; lane W, unbound proteins after His-Trap chromatography; lane P, pure protein after His-Trap chromatography. (A) Recombinant *Tt*ArsM C77S. (B) Recombinant *Tt*ArsM H40A. (C) Recombinant *Tt*ArsM H179A. Download FIG S3, TIF file, 0.6 MB. Copyright © 2021 Gallo et al. 2021 Gallo et al. https://creativecommons.org/licenses/by/4.0/ This content is distributed under the terms of the Creative Commons Attribution 4.0 International license.

### *Tt*SmtB interacts with *Tt*ArsM and binds to its promoter.

**FIG 3:**
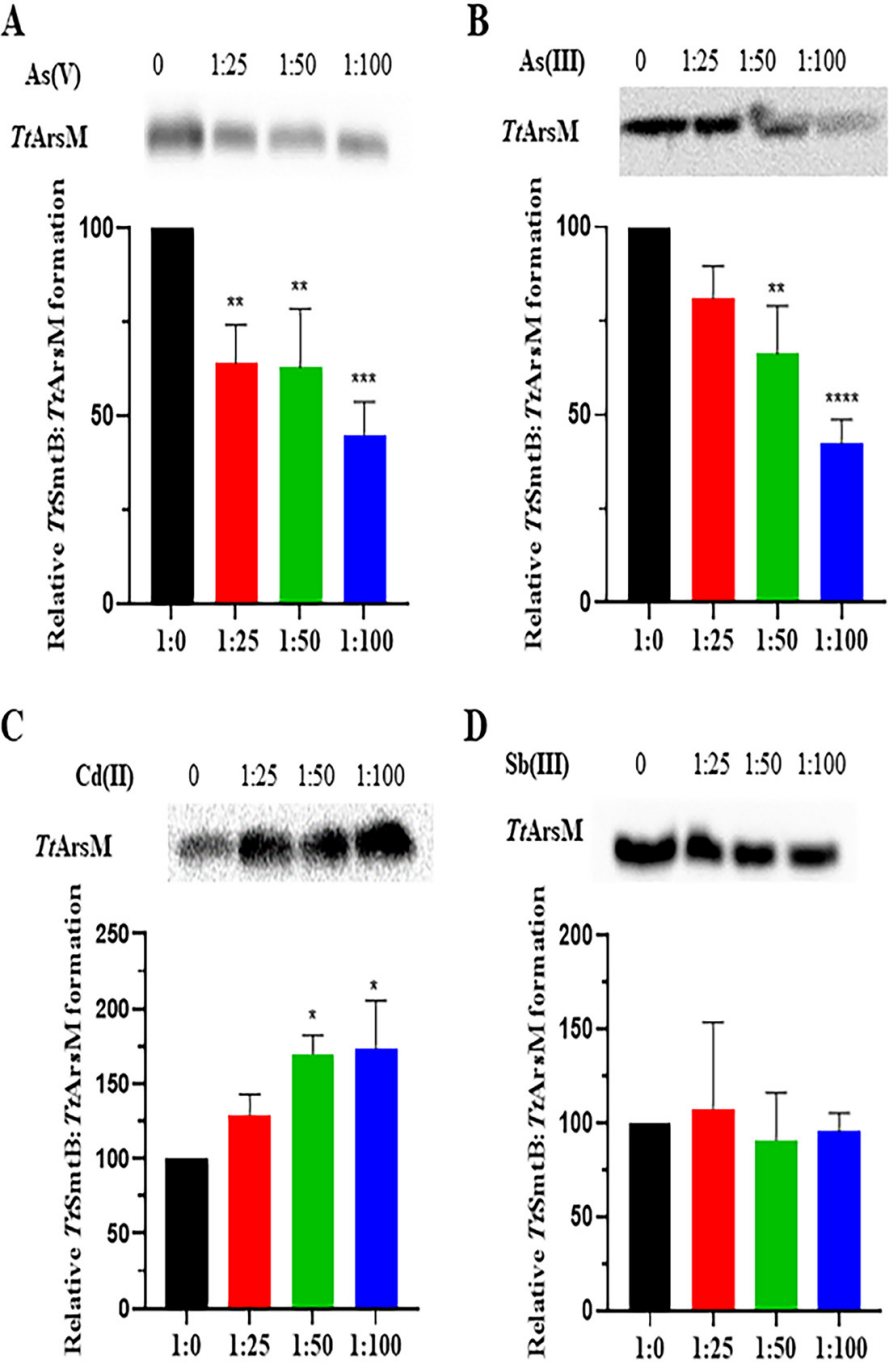
*Tt*SmtB-*Tt*ArsM interaction in the presence of heavy metal ions. Co-IP of *Tt*SmtB-*Tt*ArsM complex with increasing concentrations of arsenate (A), arsenite (B), cadmium (C), and antimony (D). Complexes were immunoprecipitated with anti-*Tt*SmtB antibodies and revealed through Western blotting using anti-His antibodies against the His tag of *Tt*ArsM. (C and D, Bottom) Densitometric analysis of blots of *Tt*SmtB:*Tt*ArsM complex. The intensity of the unchallenged complex was used as a reference. Average values from three biological replicates are shown, with error bars representing standard deviations. Statistical analysis was performed using one-way ANOVA; significant differences are indicated as *, *P* < 0.05; **, *P* < 0.01; ***, *P* < 0.001; and ****, *P* < 0.0001.

This is the first study to report the protein-protein interaction of an ArsR/SmtB transcriptional regulator with a member of the arsenic detoxification system as identified by pulldown and mass spectrometry. For this reason, we decided to confirm the physical interaction between *Tt*SmtB and *Tt*ArsM and to investigate the effect of different metals on the *Tt*SmtB-*Tt*ArsM interaction. A coimmunoprecipitation (co-IP) assay was carried out upon incubation of purified *Tt*ArsM and *Tt*SmtB, either in the presence or in the absence of arsenite, arsenate, cadmium, and antimony. The first three ions are *Tt*SmtB effectors, as their interaction weakened the binding to target promoters, while antimony had no effect on DNA recognition (32). Immunoprecipitation with anti-*Tt*SmtB antibodies, followed by detection of the His-tagged *Tt*ArsM by anti-His-tag antibodies, showed that the two proteins interact and form a complex in the absence of arsenite and arsenate, confirming the existence of physical interaction between them (Fig. 3). No band was detected when the immunoprecipitation was carried out with the unrelated control protein, *Tt*GalA (40). Increasing arsenate and arsenite concentrations negatively affected the stability of *Tt*SmtB-*Tt*ArsM complex; in fact, densitometric analysis of the Western blot revealed up to a 3-fold decrease in the intensity of the band corresponding to the complex (at 1:100 protein/arsenic ratio) in the presence of both arsenate (Fig. 3A) and arsenite (Fig. 3B). Interestingly, the presence of cadmium had the opposite effect, enhancing the band intensity by up to 2-fold (Fig. 3C), suggesting that the interaction of this metal with the complex occurs with a different mechanism. Finally, the presence of antimony had a negligible effect on complex stability, in agreement with previous data showing that this metal ion is not an effector for *Tt*SmtB (32) (Fig. 3D).

**FIG 4:**
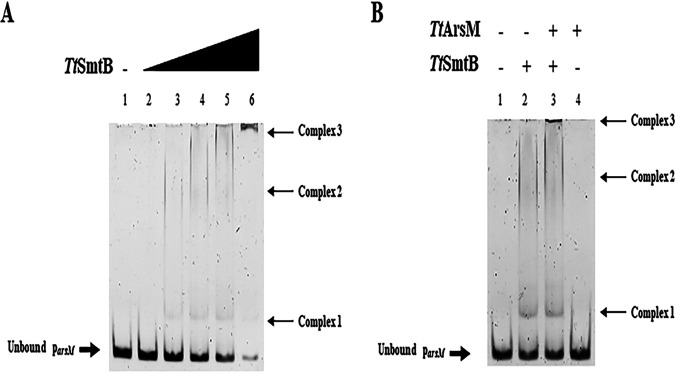
*Tt*SmtB-*p_arsM_* interaction. (A) Interaction of *p_arsM_* in the presence of increasing concentrations of *Tt*SmtB. Lane 1, negative control; lane 2, 1 μM *Tt*SmtB; lane 3, 2 μM *Tt*SmtB; lane 4, 3 μM *Tt*SmtB; lane 5, 5 μM *Tt*SmtB; and lane 6, 10 μM *Tt*SmtB. (B) EMSA with *p_arsM_* in the presence of 3 μM *Tt*SmtB and 3 μM *Tt*ArsM.

Since *Tt*SmtB is the transcriptional repressor of the genes involved in arsenic and cadmium resistance in T. thermophilus HB27 (22, 23), we hypothesized that it could also regulate *TtarsM* transcription. Sequence analysis of *TtarsM* promoter (*p_arsM_*), a 108-bp-long region upstream of *TtarsM* and encompassing the translation start codon revealed the presence of an inverted repeat region [GAAC(N14)CTTG] between positions −6 and −27 upstream of the start codon. The sequence overlaps −10 and −35 putative basal promoter region, is 100% identical to the *TtarsX* operator recognized by *Tt*SmtB, and matches the consensus binding sites of ArsR/SmtB proteins (41). Hence, we performed electrophoretic mobility shift assay (EMSA) to investigate the capacity of purified *Tt*SmtB to bind to the promoter region of *TtarsM*. *Tt*SmtB binds to *p_arsM_* in a concentration-dependent manner, as shown by the gradual formation of lower-mobility complexes and the gradual decrease of residual unbound DNA (Fig. 4A, lanes 2 to 5); at 10 μM protein, the complex hardly enters the gel, suggesting the formation of multiple dimers associated with target DNA (Fig. 4A, lane 6). This observation suggests that by interacting with the regulatory region, *Tt*SmtB controls *TtarsM* transcription in a way comparable to that already reported for other arsenic resistance genes, i.e., the arsenate reductase, the metal ion transporter, and itself (22, 23).

Since the existence of a physical interaction between *Tt*SmtB and *Tt*ArsM was established, we asked whether *Tt*ArsM influenced *Tt*SmtB interaction with *p_arsM._* Therefore, we preincubated 3 μM of both proteins before performing an EMSA under the same conditions described above. Interestingly, when the two proteins are coincubated, shifted bands can be observed (Fig. 4B, lane 3, complex 3) corresponding to complexes of higher molecular weight in comparison to those generated or not by *Tt*SmtB or *Tt*ArsM, respectively (Fig. 4B, lane 2, complex 2, and lane 4); this analysis indicates that *Tt*SmtB-*Tt*ArsM multimeric complexes bind to the promoter and suggests that *Tt*SmtB-*Tt*ArsM protein-protein interaction may function in either transcriptional and posttranscriptional control. Notably, very few studies in bacteria report protein-protein interactions of transcriptional regulators with the product of the genes they regulate (42, 43).

### *In vivo* activity of *Tt*ArsM and its mutants in E. coli.

**FIG 5:**
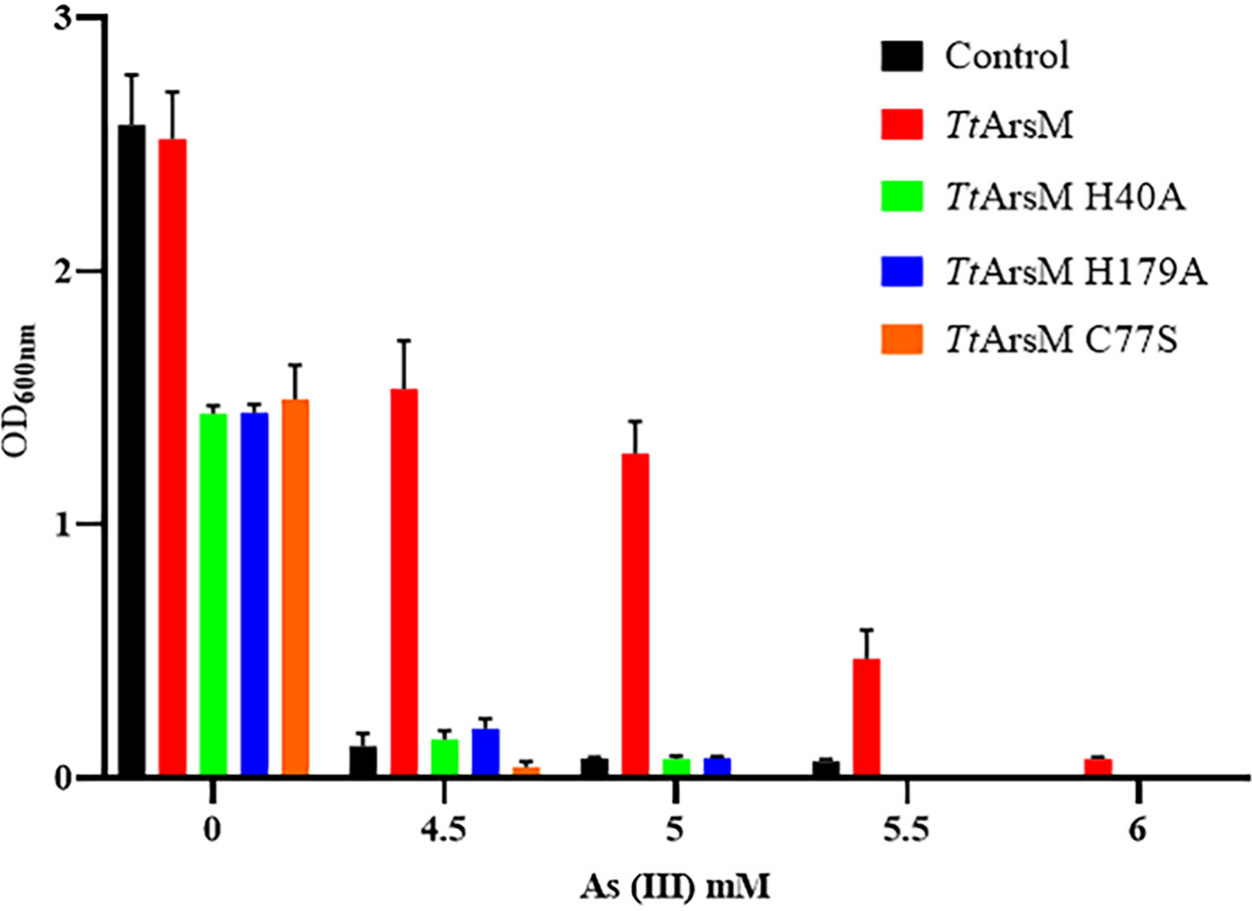
Growth of E. coli BL21 strains expressing *Tt*ArsM and its mutants, in the presence of different arsenite concentrations, measured 24 h postinoculation. The strains are E. coli BL21/pET30b (black), E. coli BL21/pET30/*Ttars*M (red), E. coli BL21/pET30/*Ttars*M H40A (green), E. coli BL21/pET30/*Ttars*M H179A (blue), and E. coli BL21/pET30/*TtarsM* C77S (orange). Average values from three biological replicates are shown, with error bars representing standard deviations.

Aiming to explore the role of *Tt*ArsM in arsenite resistance *in vivo*, we challenged E. coli BL21-CodonPlus (DE3)-RIL strains transformed with plasmids expressing *Tt*ArsM and its catalytic mutants (*Tt*ArsM C77S, *Tt*ArsM H40A, and *Tt*ArsM H179A) to grow in the presence of arsenite. Each recombinant strain was grown in the presence of different arsenite concentrations for 24 h to determine the MIC toward the metal ion. The *Tt*ArsM-expressing strain appeared to be more resistant to arsenite than the control strain (MICs of 6 mM and 4.5 mM, respectively). Additionally, the strains expressing mutated *Tt*ArsM were inhibited by the presence of arsenite to the same extent as the control strain (Fig. 5). This shows that the heterologous expression of *Tt*ArsM in E. coli increases arsenite resistance even at mesophilic temperatures, indicating the role of *Tt*ArsM in arsenite detoxification. Moreover, the result obtained with the mutant strains demonstrates the role of C77, H40, and H179 in the catalytic function of *Tt*ArsM.

### Developing a hyperthermoactive-Cas9 editing tool.

We further aimed to investigate *in vivo* the contribution of *Tt*ArsM to the arsenite detoxification mechanism via the deletion of the *TtarsM* gene from the T. thermophilus HB27 genome. Nonetheless, the currently available genome editing tool for T. thermophilus is time-consuming, not marker-free, and not always efficient (44). For this purpose, we reasoned to develop a marker-free, plasmid-based, homologous recombination (HR) Cas9 counterselection (CS) genome-editing tool for T. thermophilus employing ThermoCas9, a thermotolerant and thermoactive Cas9 orthologue (45).

**FIG 6:**
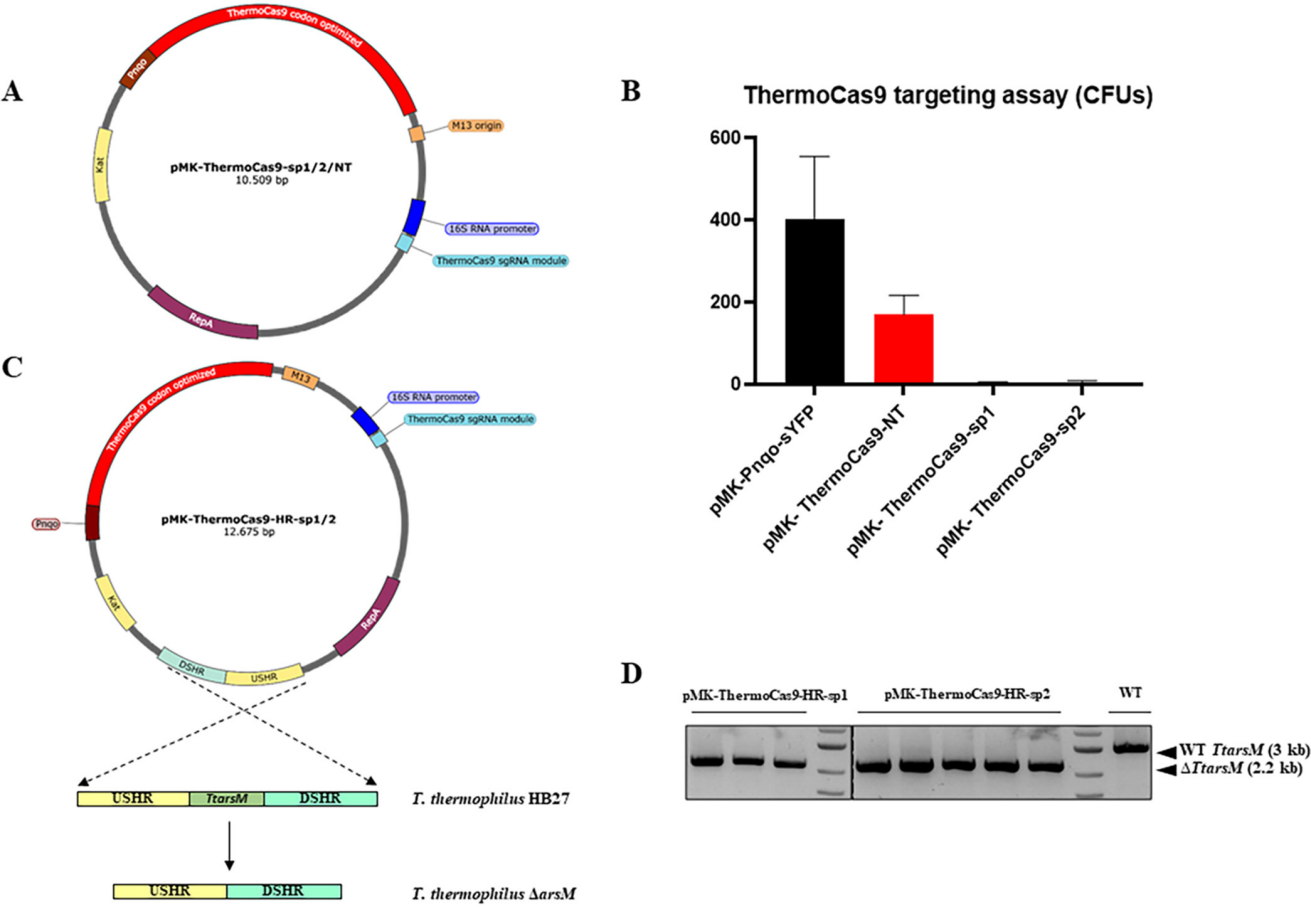
ThermoCas9-based genome engineering in T. thermophilus HB27. (A) pMK-ThermoCas9-sp1/2/NT targeting vectors. (B) Graphical representation of the ThermoCas9 targeting assay results (CFU) for assessing the ThermoCas9 toxicity and targeting efficiency in T. thermophilus HB27. Average values from three biological replicates are shown, with error bars representing standard deviations. (C) pMK-ThermoCas9-HR-sp1/2 editing vectors, employed for the genomic deletion of the *TtarsM* gene. (D) Agarose gel electrophoresis showing the resulting products from genome-specific colony PCRs on T. thermophilus colonies formed from the ThermoCas9-based *TtarsM* deletion process. A wild-type colony was subjected to the same PCR, and the related product is shown here as negative control for *TtarsM* deletion. The expected sizes of the PCR amplification products that correspond to the wild-type and Δ*TtarsM* genotypes are indicated with black arrows.

We initially evaluated the targeting efficiency of ThermoCas9 in T. thermophilus HB27. Therefore, a set of 3 vectors was constructed, namely, pMK-ThermoCas9-NT, pMK-ThermoCas9-sp1, and pMK-ThermoCas9-sp2 (Fig. 6A) by cloning into the pMK18 vector (46) (i) the codon-harmonized version of the *thermocas9* gene under the transcriptional control of the constitutive *nqo* promoter (44), and (ii) the single guide RNA (sgRNA)-expressing module under the transcriptional control of the constitutive 16S rRNA promoter, either with a nontargeting/control spacer (NT, 5′-CTAGATCCGCAGTAACCCCATGG-3′) or with spacers that target the *TtarsM* gene (sp1, 5′-GGGCGTTGGTGATGTGGGCCCTC-3′, and sp2, 5′-CCACCTCCTCCTCCCGGTAAGGC-3′). The 3 vectors were used to transform T. thermophilus HB27, along with pMK-Pnqo-syfp vector (27), as transformation control. The cells were allowed to recover at 70°C before being plated on selective agar plates and incubated overnight at 60°C due to the sensitivity of pMK18 at temperatures above 65°C. The transformation efficiencies of pMK-ThermoCas9-sp1 and pMK-ThermoCas9-sp2 targeting vectors were significantly reduced compared to the transformation efficiency with the pMK-ThermoCas9-NT nontargeting vector (Fig. 6B). Moreover, the transformation efficiency with the pMK-Pnqo-syfp vector was only slightly higher than the transformation efficiency with the pMK-ThermoCas9-NT vector (Fig. 6B), which could be attributed to the significant size difference between the two vectors (7,552 bp and 13,554 bp, respectively). This result indicates that ThermoCas9 is expressed in T. thermophilus HB27 cells in an active and not-toxic form, motivating the development of a ThermoCas9-based genome-editing tool.

We set out to develop and test the efficiency of an HR ThermoCas9-based CS genome-editing tool in T. thermophilus HB27. For this purpose, we introduced an HR template for the deletion of the *TtarsM* gene into the 3 previously described ThermoCas9 vectors. The HR template was composed of the fused 1-kb upstream and downstream flanking regions of the *TtarsM* gene (Fig. 6C). The three resulting editing vectors, namely, pMK-ThermoCas9-HR-NT, pMK-ThermoCas9-HR-sp1, and pMK-ThermoCas9-HR-sp2, were transformed into T. thermophilus HB27 cells, recovered at 70°C, and grown on selective agar plates overnight at 60°C. Colony PCR with genome-specific primers was subsequently employed to screen several colonies for each transformation (Fig. S4; Table S2). None of the colonies from the pMK-ThermoCas9-NT transformation were clean Δ*TtarsM* mutants (0/10 colonies), and only a small number of colonies were mixed wild-type/Δ*TtarsM* mutants (2/10 colonies) (Fig. S4A; Table S2). On the other hand, almost all the screened colonies from the pMK-ThermoCas9-sp1 transformation were clean Δ*TtarsM* mutants (19/19 colonies) (Fig. S4B; Table S2); most of the screened colonies from the pMK-ThermoCas9-sp2 transformation were clean Δ*TtarsM* mutants (13/18 colonies), and the remaining were mixed wild-type/Δ*TtarsM* mutants (5/18 colonies) (Fig. S4C; Table S2); the latter result suggests that less efficient ThermoCas9 targeting is obtained when employing spacer 2. Subsequently, DNA sequencing on randomly selected clean Δ*TtarsM* mutant colonies was performed to verify the correctness of the genome editing (Fig. S4D; Table S2).

10.1128/mBio.02813-21.2 TABLE S2 Results from genome-specific colony PCRs on T. thermophilus HB27 colonies formed from the ThermoCas9-based *TtarsM* deletion experiments. Download Table S2, DOCX file, 0.02 MB. Copyright © 2021 Gallo et al. 2021 Gallo et al. https://creativecommons.org/licenses/by/4.0/ This content is distributed under the terms of the Creative Commons Attribution 4.0 International license.

10.1128/mBio.02813-21.9 FIG S4 Agarose gel electrophoresis from genome-specific colony PCRs on T. thermophilus HB27 colonies formed from the ThermoCas9-based *TtarsM* deletion process upon transformation with the pMK-ThermoCas9-HR-NT control-editing vector (A), the pMK-ThermoCas9-HR-sp1-editing vector (B), and the pMK-ThermoCas9-HR-sp2-editing vector (C). Wild-type colonies were subjected to the same PCR, and the related products are shown as negative controls for *TtarsM* deletion. The expected sizes of the PCR amplification products that correspond to the wild-type and Δ*TtarsM* genotypes are indicated with arrows. (D) Sequencing chromatogram of the PCR-amplified *TtarsM* genomic region from a randomly selected colony, previously PCR screened as Δ*TtarsM.* (E) Agarose gel electrophoresis after the curing process of the editing vectors shows the absence of products using pMK18-specific primers for colony PCRs on T. thermophilus HB27 Δ*TtarsM* colonies. A wild-type colony containing the pMK18 vector was subjected to the same PCR, and the related product is shown here as a negative control of the curing process. Download FIG S4, TIF file, 0.5 MB. Copyright © 2021 Gallo et al. 2021 Gallo et al. https://creativecommons.org/licenses/by/4.0/ This content is distributed under the terms of the Creative Commons Attribution 4.0 International license.

Aiming to test the temperature limit of the developed ThermoCas9-based genome-editing tool, we repeated the editing experiment, increasing the plating temperature to 65°C, corresponding to the temperature limit of the pMK18 backbone for propagation. Under these conditions, the numbers of colonies formed upon transformation with the pMK-ThermoCas9-sp1 and pMK-ThermoCas9-sp2 were 3 and 5 vectors, respectively, much lower than the corresponding numbers when the plating temperature was 60°C (Table S2). This can be ascribed to the high ThermoCas9 targeting activity at 65°C (45) and decreased vector stability at 65°C. Nonetheless, the DNA sequence of all the screened colonies confirmed that they were clean Δ*TtarsM* mutants, demonstrating the high efficiency of the developed tool at 65°C (Fig. 6D).

Finally, assuming that the curing of the editing plasmid from a Δ*TtarsM* mutant strain would facilitate additional editing steps, we randomly selected a Δ*TtarsM* mutant colony for inoculation in liquid antibiotic-free TM medium for two culturing rounds at 65°C and then plated the cultures on TM agar plates with and without antibiotic. Multiple colonies were found on the antibiotic-free plate and no colonies on the plate supplemented with the antibiotic, demonstrating that the cells were cured from the edited vector. Seven of these colonies were randomly selected and the absence of the plasmid confirmed by colony PCR using *thermoCas9-*specific primers (Fig. S4E).

Therefore, a markerless HR-ThermoCas9-based CS genome-editing tool was developed for T. thermophilus HB27, highly efficient at temperatures up to 65°C. Using this tool, a T. thermophilus Δ*TtarsM* strain was constructed in less than 10 days (including the plasmid-curing process), expanding the repertoire of available genetic tools for this microorganism and considerably accelerating the required time for editing its genome.

### *Tt*ArsM mutant is more sensitive to arsenite.

**FIG 7:**
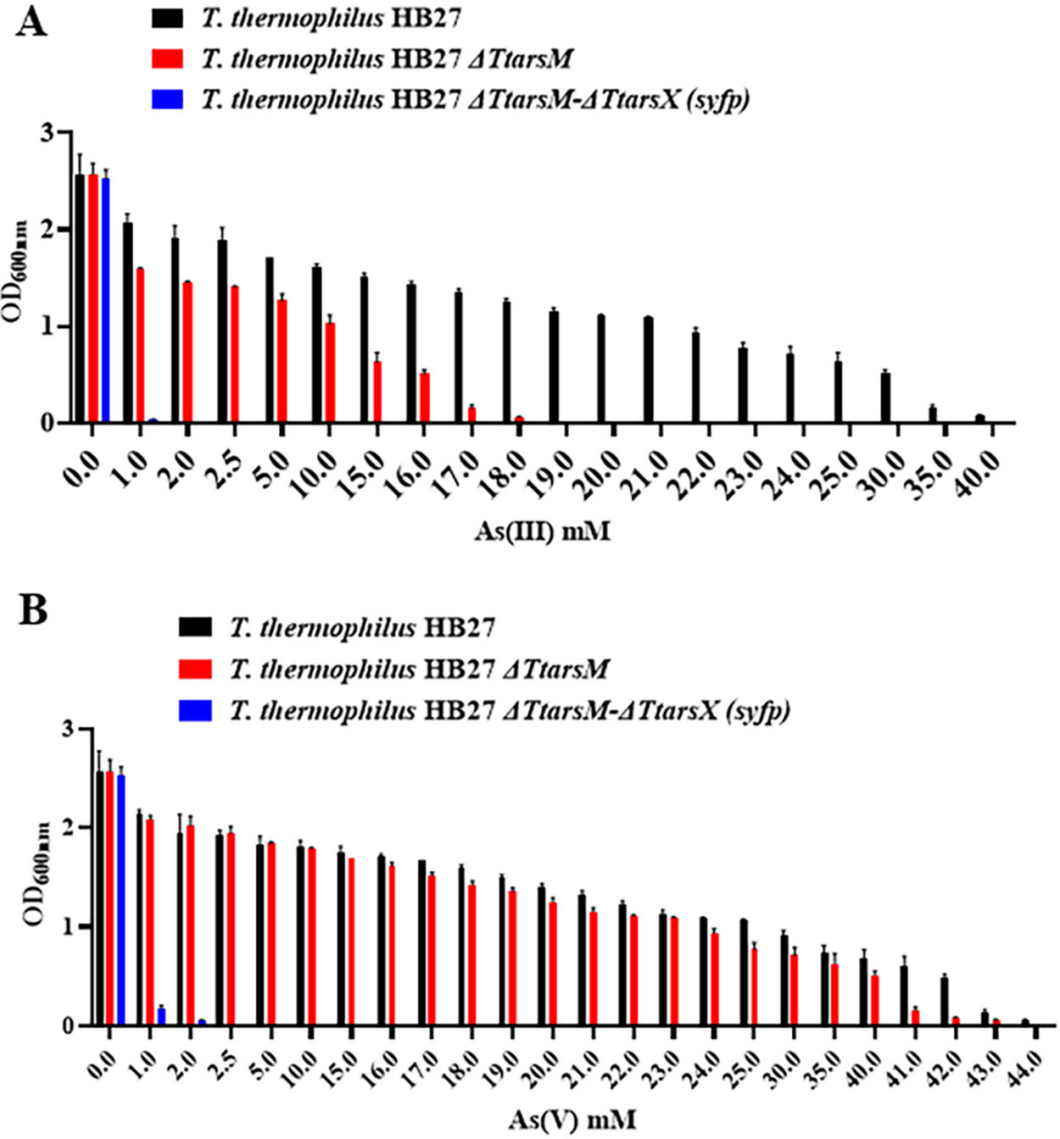
Growth of T. thermophilus HB27 (black), T. thermophilus HB27 Δ*TtarsM* (red), and T. thermophilus HB27 Δ*TtarsM-*Δ*TtarsX* (*syfp*) (blue) in TM medium in the presence of different concentrations of arsenite (A) and arsenate (B) measured 24 h after inoculation. Average values from three biological replicates are shown, with error bars representing standard deviations.

To compare the arsenic resistance of Δ*TtarsM* to that of wild-type T. thermophilus HB27, both strains were grown in TM liquid medium with different arsenite and arsenate concentrations for 24 h (Fig. 7). As expected, the arsenite resistance of Δ*TtarsM* was significantly lower than that of the wild-type strain, with the corresponding MIC values being 18 mM and 40 mM, respectively (Fig. 7A). Moreover, the resistance of the Δ*TtarsM* strain to arsenate was comparable to the wild-type strain (42 mM and 44 mM, respectively) (Fig. 7B), in agreement with its role in arsenite resistance. This result confirmed that the thermoactive arsenite methyltransferase *Tt*ArsM is involved in arsenite detoxification and is a novel component of the arsenic resistance machinery.

### Developing a sensitive arsenic bioreporter.

In a previous study, we demonstrated that *Tt*ArsX is the arsenic efflux membrane protein of T. thermophilus HB27 and reported that a Δ*TtarsX* mutant strain is more sensitive to arsenate and arsenite (23). In this study, we wanted to ascertain whether a strain lacking both *TtarsM* and *TtarsX* would be even more sensitive to arsenic ions than the single-mutant Δ*TtarsX* and Δ*TtarsM* strains and therefore could represent an even better bioreporter strain for arsenic detection.

For this purpose, the HR ThermoCas9-based CS-editing tool was employed to exchange the *TtarsX* gene on the genome of the Δ*TtarsM* strain with the *syfp* reporter gene (27), setting the expression of the encoded thermotolerant yellow fluorescence protein (sYFP) under the control of the arsenic-responsive *TtarsX* promoter (*p_arsX_*). The employed editing vector, denoted as pMK-ThermoCas9-HR-*syfp*, contained a spacer that targets the *TtarsX* gene (5′-TTTCGACGGAGGAGGCCTTGGCC-3′) and an HR template composed of the 1-kb upstream flanking genomic region of *TtarsX* followed by *syfp* and the 1-kb downstream flanking genomic region of *TtarsX*. Ten colonies grown after transformation of pMK-ThermoCas9-HR-*syfp* vector into T. thermophilus Δ*TtarsM* cells were screened by colony PCR with genome-specific primers and sequenced; eight of them were clean T. thermophilus HB27 Δ*TtarsM-*Δ*TtarsX* (*syfp*) knock-in mutants (Fig. S5), also proving that the developed tool was highly efficient for gene insertions and substitutions.

10.1128/mBio.02813-21.10 FIG S5 Agarose gel electrophoresis showing the resulting products from genome-specific colony PCRs on T. thermophilus HB27 Δ*TtarsM-*Δ*TtarsX (syfp)* colonies formed from the ThermoCas9-based substitution process of the *TtarsX* gene by the *syfp* gene. A wild-type colony was subjected to the same PCR, and the related product is shown as a negative control for *TtarsX* substitution. The expected sizes of the PCR amplification products that correspond to the wild-type and Δ*TtarsX* (*syfp*) genotypes are indicated with black arrows. Eight out of the 10 screened clones were T. thermophilus Δ*TtarsM-*Δ*TtarsX* (*syfp*) knock-in mutants. Download FIG S5, TIF file, 0.3 MB. Copyright © 2021 Gallo et al. 2021 Gallo et al. https://creativecommons.org/licenses/by/4.0/ This content is distributed under the terms of the Creative Commons Attribution 4.0 International license.

The double-mutant strain was challenged with different arsenite and arsenate concentrations in TM liquid medium. As shown in Fig. 7A and B, arsenite resistance is strikingly lower (0.5 mM) than that of the wild-type (40 mM), Δ*TtarsM* (18 mM), and Δ*TtarsX* strains (3 mM) (23). Interestingly, the Δ*TtarsM-*Δ*TtarsX* (*syfp*) strain showed also lower resistance to arsenate than the single-mutant *ΔTtarsX* (2 mM and 3 mM, respectively) (22).

**FIG 8:**
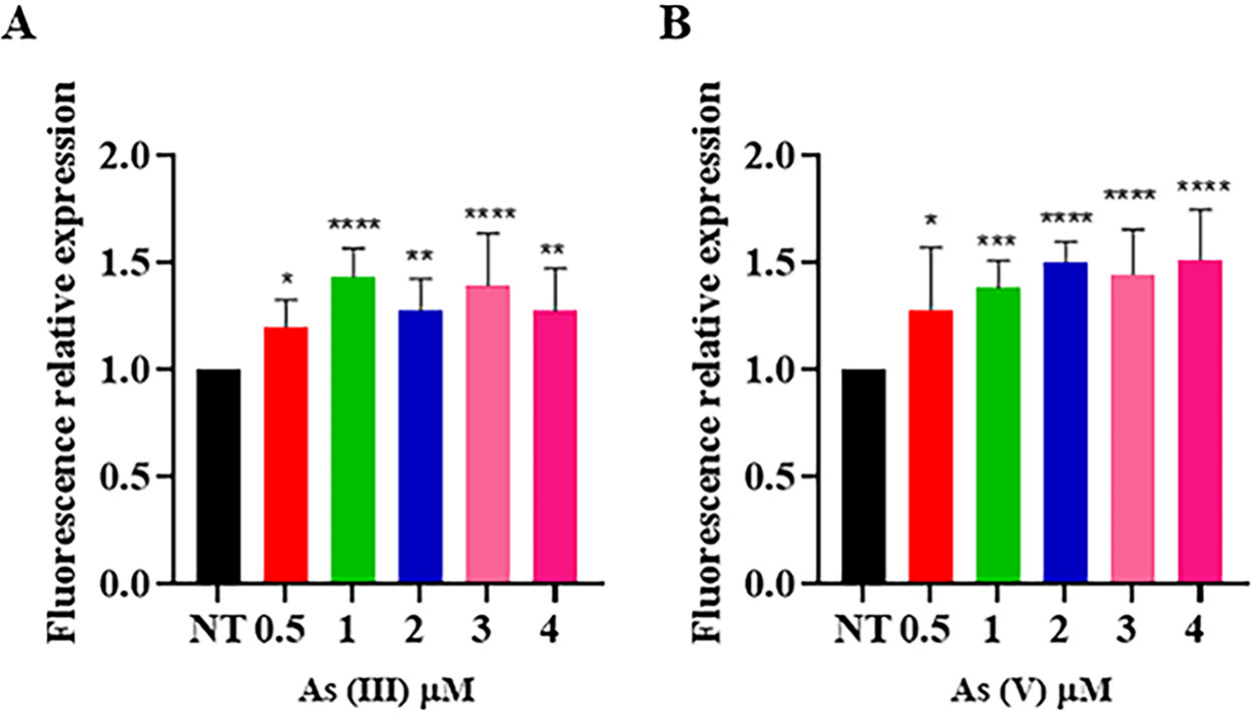
Bioreporter activity. T. thermophilus HB27 Δ*TtarsM-*Δ*TtarsX* (*syfp*) bioreporter strain challenged with increasing concentrations of arsenite (A) and arsenate (B). Average values from three biological replicates are shown, with error bars representing standard deviations. Statistical analysis was performed using one-way ANOVA; significant differences are indicated as *, *P* < 0.05; **, *P* < 0.01; ***, *P* < 0.001; and ****, *P* < 0.0001.

To evaluate the sensitivity to arsenate and arsenite of the whole-cell bioreporter system, exponentially growing cultures of T. thermophilus HB27 Δ*TtarsM-*Δ*TtarsX* (*syfp*) were treated with increasing concentrations of arsenite and arsenate, and the intensity of the emitted fluorescence was compared (Fig. 8). The background fluorescence of the Δ*TtarsM-*Δ*TtarsX* (*syfp*) strain was low, indicating that the system is repressed in the absence of metal ions. Moreover, the developed bioreporter system was able to detect arsenite and arsenate concentrations as low as 0.5 μM (Fig. 8). This performance substantially overtakes the detection limit of the previously developed arsenite and arsenate bioreporter system, which was based on the T. thermophilus Δ*TtarsX* strain and plasmid-based expression of the β-galactosidase (23).

## DISCUSSION

In this study, we aimed to identify novel proteins involved in the arsenic resistance system of T. thermophilus HB27 and employed *Tt*SmtB as a starting point looking beyond its transcriptional regulation activity. As *Tt*SmtB contains a protein interaction domain (32), we set out to identify putative *Tt*SmtB-interacting proteins with a role in arsenic metabolism/detoxification, following an immunoprecipitation and comparative proteomics approach. This strategy led to the discovery of *Tt*ArsM, the first prokaryotic thermoactive arsenite SAM-dependent methyltransferase, evolutionarily distant from other known arsenite methyltransferases.

The original structure and activity mechanism of *Tt*ArsM were explored *in silico*. Like other arsenite methyltransferases known to date, *Tt*ArsM contains a C-terminal SAM-dependent methyltransferase domain and an N-terminal domain comprised of only one (instead of the usual three) conserved cysteine; the other two catalytic cysteines required for arsenite coordination might be replaced by two histidines identified by docking analysis. Interestingly, H40 is part of an arsenite binding domain typical of many ArsR metalloregulatory proteins (47), and H179 is located close to the cysteine (C177) of the arsenite methyltransferase of the extremophilic alga Cyanidioschyzon merolae (48). Moreover, the conservation of these three amino acids among the putative arsenite methyltransferases in the *Thermus* genus supports their possible role in catalysis and suggests an adaptation in this group of microorganisms.

Indeed, the role of the cysteine and histidine residues in the structure-function of *Tt*ArsM predicted *in silico* was demonstrated by site-directed mutagenesis, as the heterologous expression in E. coli of *Tt*ArsM mutants conferred lower arsenite resistance than *Tt*ArsM.

To date, only for a few arsenite methyltransferases, the reaction mechanism has been reported (13, 20, 38, 39, 49). Those described possess three, or at least two, cysteine residues present in their catalytic site that can methylate the arsenic sequentially in its trivalent form through alternating reduction and oxidative methylation reactions; of note, different enzymes produce mono-, di-, and trimethylated forms of arsenic in diverse amounts, highlighting a biochemical diversity in the arsenite methylation mechanism (38). The newly discovered *Tt*ArsM, highly conserved within the *Thermus* genus, possesses only one cysteine and can methylate As(III) mainly into DMAs and a smaller amount of MMAs as determined by GC-MS analysis of the products of the *in vitro* assay. To the best of our knowledge, this is the first arsenite methyltransferase functioning with a single cysteine in the active site.

The discovered transcriptional and posttranslational interaction of *Tt*ArsM with the transcriptional regulator *Tt*SmtB was investigated in more detail. It was demonstrated that *Tt*SmtB binds to the promoter region of *TtarsM* and that this binding is stabilized by the *Tt*SmtB-*Tt*ArsM complex. Moreover, co-IP experiments confirmed the interaction of *Tt*SmtB with *Tt*ArsM and showed a reverse correlation between the stability of the complex and arsenic concentration. Presumably, the complex enhances the repression of *TtarsM* transcription in the absence of arsenic ions through a novel mechanism. Hence, through this analysis, we shed light on a novel kind of interaction, rarely described for bacteria, in which the transcriptional repressor of a gene interacts with the protein product of the gene that it regulates (43, 50).

An example of an enzyme that can modulate the transcriptional activity of regulators by protein-protein interaction is reported in the cysteine metabolism of Bacillus subtilis where the stable complex formed by CymR (the master regulator of the system) and CysK (*O*-acetyl-l-serine-thiol-lyase) represses the transcription of the genes involved in the cysteine pathway (including the *cysK* gene itself) when cysteine concentration is low. The advantage of this regulatory mechanism is that it employs enzymes that can specifically recognize their substrates or allosteric effectors; thus, enzymes and/or transcriptional regulators can act simultaneously as intracellular molecular sensors and participate in their own transcriptional regulation (42).

The role of *Tt*ArsM in T. thermophilus HB27 arsenic detoxification was also demonstrated via the construction and characterization of a *ΔTtarsM* mutant strain. For this purpose, a marker-free homologous recombination and ThermoCas9-based counterselection genome-editing tool was developed which was highly efficient and active at temperatures up to 65°C. Our tool equals the highest reported temperature for a Cas9-based editing tool to date (51, 52). The characterization of the T. thermophilus *ΔTtarsM* strain confirmed its expected higher sensitivity to arsenite, but not arsenate, than the wild-type strain. To better define the role of *Tt*ArsM in the context of the already characterized components of the arsenic resistance system, a double mutant was constructed upon exchanging the *TtarsX* efflux pump gene in the T. thermophilus Δ*TtarsM* genome with the gene encoding the yellow fluorescent protein; the double mutant resulted as much more sensitive to arsenite and arsenate treatment. Hence, it was demonstrated that *Tt*ArsM and *Tt*ArsX are critical players of the arsenite detoxification system. This is the first example of a successful insertion of a heterologous gene on the T. thermophilus genome by genome editing. The double-mutant strain was also considered a sensitive bioreporter for the development of a whole-cell biosensor system. Indeed, it was able to detect arsenite and arsenate concentrations as low as 0.5 μM, showing 40 times higher sensitivity than the previously developed T. thermophilus HB27 *ΔTtarsX*- plasmid-based biosensor (23).

In conclusion, this study explores a unique strategy to identify novel enzymes and/or regulative networks in nonmodel bacteria and expands the repertoire of genetic systems for hyperthermophiles. In addition, this work has resulted in a gain of insight into the arsenite/arsenate detoxification mechanism, particularly that of T. thermophilus. On top of that, this has allowed us to develop a highly robust and sensitive biosensor.

## MATERIALS AND METHODS

### T. thermophilus HB27 cell extract preparation.

T. thermophilus HB27 cultures were grown aerobically at 70°C in TM medium, as previously described (24). Once the cultures reached an optical density at 600 nm (OD_600_) of 0.5, they were treated either with 8 mM NaAsO_2_ or with 12 mM NaH_2_AsO_4_ (Sigma) (the used concentrations were below the previously reported MIC values for arsenate and arsenite) (22, 23) or they remained untreated. Samples were harvested from each culture, either immediately after treatment or 60 min posttreatment. The samples were centrifuged, the precipitates were resuspended in phosphate buffer (20 mM Na_3_PO_4_, pH 7.5) supplemented with protease inhibitor cocktail (Thermo Scientific), and the resuspended cells were lysed by sonication (10 cycles of 30 s on/30 s off, 40% power; Misonix Sonicator Ultrasonic Processor XL). The lysates were centrifuged and the cell extracts (CFE) used for pulldown assays.

### Purification of recombinant *Tt*SmtB, immobilized metal affinity chromatography, and pulldown.

C-terminal His-tagged *Tt*SmtB was purified from E. coli BL21-CodonPlus (DE3)-RIL cells transformed with the pET28/*Ttsmt*B vector, as previously described (22). Purified C-terminal His-tagged *Tt*SmtB (2 mg) was incubated with 200 μl of Ni^2+^-NTA resin (Sigma-Aldrich) equilibrated in 20 mM Na_3_PO_4_, 0.5 M NaCl, and 20 mM imidazole, pH 7.5, for 16 h at 4°C and then washed three times with the same buffer to remove unbound proteins. T. thermophilus HB27 CFE, treated with arsenite, treated with arsenate, or not treated were incubated with the functionalized resin (Ni^2+^-NTA/*Tt*SmtB) for 16 h at 4°C under stirring conditions. Subsequently, the resin was extensively washed, and the interacting proteins were eluted with 20 mM Na_3_PO_4_, 0.5 M NaCl, and 0.5 M imidazole, pH 7.5. As negative controls, samples of Ni^2+^-NTA resin not functionalized with *Tt*SmtB were incubated with the same T. thermophilus HB27 CFE.

### *In situ* hydrolysis and LC-MS/MS analysis.

The fractions eluted from the pulldown process were analyzed by 15% SDS-PAGE and *in situ* hydrolyzed for mass spectrometry analysis. Specifically, monodimensional SDS-PAGE gel was colored with Coomassie brilliant blue; the revealed bands were cut and destained with 100 μl of 0.1 M ammonium bicarbonate (AMBIC) and 130 μl of acetonitrile (ACN). Each band was hydrolyzed *in situ* with 0.1 μg/μl trypsin in 10 mM AMBIC and incubated at first for 1.5 h at 4°C and then for an additional 16 h at 37°C. The hydrolysis reactions were stopped by adding acetonitrile and 0.1% formic acid; then, the samples were filtered and dried in a Savant vacuum centrifuge before being analyzed by LC-MS/MS mass spectrometry. In detail, before analysis, the samples were dissolved in 10 μl of 0.1% formic acid, and 5 μl were directly loaded into the instrument. Reverse-phase capillary liquid chromatography (HPLC 1200 system experiments), followed by MS analysis, was performed using a binary pump system connected to a nanospray source of the mass spectrometer (28, 53). The latter is represented by a hybrid quadrupole time of flight (Q-TOF) spectrometer (MS Chip 6520 QTOF) equipped with a chip (Agilent Technologies).

### *In silico* analysis.

Analysis of the LC-MS/MS data, using Mascot software (http://www.matrixscience.com/search_form_select.html), allowed the identification of putative *Tt*SmtB-interacting proteins. Among these proteins, TTC0109 (UniProt code Q72LF0), here named *Tt*ArsM, was further analyzed using the UniProt database (http://www.uniprot.org); homologous proteins and conserved domains were identified by performing a BLAST analysis (https://blast.ncbi.nlm.nih.gov/Blast.cgi).

A phylogenetic tree of archaeal and bacterial arsenite methyltransferases, SAM-dependent methyltransferases, and methyltransferase domain-containing proteins, including *Tt*ArsM from T. thermophilus HB27, was conducted in MEGA X (54). The amino acidic sequences used for the construction of the phylogenetic tree are *Tt*ArsM from T. thermophilus HB27; arsenite methyltransferase from Rhodopseudomonas palustris (13), Methanosarcina acetivorans, *Clostridium* sp. strain BMX (55), Halobacterium salinarum (56), Pseudomonas alcaligenes (38), and Cyanidioschyzon merolae (48); a SAM-dependent methyltransferase from 5 members of the *Thermus* genus (T. islandicus, T. caldilimi, T. antranikianii, T. oshimai, and T. brockianus); a SAM-dependent methyltransferase from Mesorhizobium amorphae and Anaerolineae bacterium; and a methyltransferase domain-containing protein from *Sanguinobacter* sp. Phylogenetic reconstruction was accomplished using the maximum-likelihood statistical method.

The alignment of *Tt*ArsM to its templates was based on a multiple-sequence alignment, performed with the program Clustal Omega (57); the amino acidic sequences used for the construction of the alignment of functionally characterized archaeal and bacterial arsenite methyltransferases are *Tt*ArsM from T. thermophilus HB27, 5 members of the *Thermus* genus (T. islandicus, T. caldilimi, T. antranikianii, T. oshimai, and T. brockianus), *Clostridium* sp. BMX, R. palustris, M. acetivorans, H. salinarum, P. alcaligenes, and Cyanidioschyzon merolae (48).

Models of *Tt*ArsM were generated through I-TASSER (58) (https://zhanglab.ccmb.med.umich.edu/I-TASSER/) using as input the complete sequence of *Tt*ArsM (C score, −2.5). The dimeric structure was predicted using the GalaxyWEB tool (http://galaxy.seoklab.org/index.html) (59). The molecular dockings of *Tt*ArsM with arsenite and SAM were generated using the Hex protein docking server (60). One hundred rigid-body docking solutions were generated per case, and the best 10 were refined by energy minimization. The proposed model for the metal ion docked into *Tt*ArsM is the structure with the smallest distance between arsenite-histidine and cysteine-SAM (4.33 Å from H40 and 5.77 Å from H179 in *Tt*ArsM model and 4.40 Å from C77).

### Cloning, expression, and purification of recombinant *Tt*ArsM and *Tt*ArsM mutants.

The pET30b(+)*/TtarsM* vector was constructed for the expression and subsequent purification of the C-terminal His-tagged version of *Tt*ArsM. For the construction of the pET30b(+)*/TtarsM*, *TtarsM* gene was PCR amplified from T. thermophilus HB27 genome, using *Taq* DNA polymerase (Thermo Fisher Scientific) and primers containing the NdeI (*arsMfw*; Table S3 in the supplemental material) and HindIII (*arsMrv*; Table S3) restriction sites at their 5′ ends. The PCR product was purified, digested with the NdeI and HindIII restriction enzymes (NEB), and ligated (T4 ligase; NEB) into NdeI/HindIII-digested pET30b(+) vector (Novagen). The ligase mixture was transformed into E. coli TOP10F′ cells, which were plated on LB agar plates supplemented with 50 μg/ml kanamycin (Sigma-Aldrich). Single colonies were selected and inoculated in LB liquid medium supplemented with 50 μg/ml kanamycin. Plasmid isolation and sequencing were subsequently performed before transforming E. coli BL21-CodonPlus (DE3)-RIL cells with pET30b(+)*/TtarsM* vector.

10.1128/mBio.02813-21.3 TABLE S3 List of the primers used in this study. Download Table S3, DOCX file, 0.02 MB. Copyright © 2021 Gallo et al. 2021 Gallo et al. https://creativecommons.org/licenses/by/4.0/ This content is distributed under the terms of the Creative Commons Attribution 4.0 International license.

To obtain mutation of *TtarsM* gene sequence at specific sites, the QuikChange II-E site-directed mutagenesis kit (Agilent Technologies) was employed; pET30b(+)*/TtarsM* was used as a template and amplified with three different mutagenic primer pairs (Table S3) to get pET30b(+)*/TtarsM* C77S, pET30b(+)*/TtarsM* H40A, and pET30b(+)*/TtarsM* H179A vectors. The reaction mixtures were transformed into E. coli TOP10F′ cells were plated on LB agar plates supplemented with kanamycin (50 μg/ml). Single colonies were randomly selected and inoculated in LB liquid medium supplemented with kanamycin (50 μg/ml). Plasmid isolation was subsequently performed, and E. coli BL21-CodonPlus (DE3)-RIL cells were transformed with sequence-verified pET30b(+)*/TtarsM* C77S, pET30b(+)*/TtarsM* H40A, and pET30b(+)*/TtarsM* H179A vectors.

For protein expression of the His-tagged versions of *Tt*ArsM C77S, *Tt*ArsM H40A, and *Tt*ArsM H179A catalytic mutants, the recombinant E. coli BL21-CodonPlus (DE3)-RIL strains were cultured in LB medium supplemented with kanamycin (50 μg/ml) and chloramphenicol (33 μg/ml). Protein expression was induced via the addition of 1 mM isopropyl-1-thio-β-d-galactopyranoside (IPTG) when the cultures reached OD_600_ of 0.7. The cultures were further incubated with vigorous shaking at 37°C for 16 h, then centrifuged, resuspended in lysis buffer (20 mM NaP, pH 7.4, 50 mM NaCl, and 20 mM imidazole) supplemented with protease inhibitor cocktail (Thermo Scientific), and lysed by sonication (10 cycles of 30 s on/30 s off, 40% power; Misonix Sonicator Ultrasonic Processor XL). The lysates were centrifuged and the supernatants used for the purification on HisTrap HP columns (1 ml; GE Healthcare) connected to an AKTA Explorer system (GE Healthcare). The fractions containing His-tagged *Tt*ArsM proteins were eluted from the columns using a linear gradient of the elution buffer (20 mM NaP, pH 7.4, 50 mM NaCl, and 500 mM imidazole). The eluted protein fractions were subjected to SDS-PAGE analysis, and the fractions containing purified *Tt*ArsM were pooled and dialyzed for 16 h at 4°C in 20 mM NaP, pH 7.4, buffer supplemented with protease inhibitor cocktail (Thermo Scientific). The identity of the purified His-tagged *Tt*ArsM protein was confirmed by mass spectrometry, and protein aliquots were stored at −20°C.

### *Tt*ArsM quaternary structure assessment.

The native molecular mass of *Tt*ArsM was determined by loading 500 μg of the purified protein onto an analytical Superdex PC75 column (3.2 by 30 cm) connected to an AKTA Pure system in 50-mM Tris-HCl, pH 7.5, and 0.2-M KCl buffer. The column was calibrated using a set of gel filtration markers (low range; GE Healthcare), including ovalbumin (43.0 kDa), carbonic anhydrase (29.0 kDa), RNase A (13.7 kDa), and aprotinin (6.5 kDa) as previously described (24).

### Methyltransferase activity assay.

According to the manufacturer's protocol, the *Tt*ArsM arsenite methyltransferase activity was measured using the SAM510 SAM methyltransferase assay kit (G-Biosciences) with modifications regarding the temperature, SAM concentration, and reaction time (33–35). The assay relies on the degradation of *S*-adenosylhomocysteine (SAH) into urate and hydrogen peroxide by a mixture of enzymes (adenosylhomocysteine nucleosidase, adenine deaminase, and xanthine oxidase). Then, the reaction of hydrogen peroxide with 4-aminoantipyrine produces 5‐dichloro‐2‐hydroxybenzene sulfonic acid (DHBS) with ε_mM_ of 15.0 at 510 nm. A typical reaction mixture containing 200 μM As(III), 800 μM SAM, 3.1 μM the enzyme, SAM enzyme mixture, and SAM colorimetric mix in a final reaction volume of 115 μl was incubated for 1 h at 50°C in a Synergy HTX multimode microplate reader (BioTek). The same reaction mixture was tested with 10 μg of *Tt*ArsC or 10 μg of *Tt*SmtB as negative controls. One unit of arsenite methyltransferase produces 1.0 μmol of DHBS per minute at 50°C under the conditions described above. Preliminary assays were performed to define substrate saturating concentrations, varying the As(III) and SAM concentrations from 50 μM to 300 μM and from 200 μM to 1.2 mM, respectively.

### *In vitro* arsenite methylation.

As(III) methylation by *Tt*ArsM was determined in an assay solution containing 10 μM *Tt*ArsM, 250 μM As(III), 6 mM glutathione (GSH), and 1 mM SAM, in Na-phosphate 50 mM, pH 7.4, at 65°C for 24 h; the same reaction without *Tt*ArsM was used as the negative control (control sample). The reactions were terminated by the addition of 10% (vol/vol) H_2_O_2_. Samples were then filtered through 0.22-μm mixed cellulose ester (MCE) syringe filters and used for GC-MS analysis performed in an Agilent GC 6890, coupled with a 5973 MS detector. A modification of the Huang protocol (36) was employed for the detection of the methylated products, and the arsenic compounds were analyzed by using hyphenated mass spectrometry techniques without derivatization. Unambiguous identification of arsenic compounds was accomplished using authentic standards, including disodium methyl arsonate hexahydrate (MMAs) (ChemService) and dimethylarsinic acids (DMAs) (Merck Life Science S.r.l.).

In more detail, 200 μl of each sample was treated with 800 μl of methanol (Sigma-Aldrich). The supernatants were recovered, vacuum-dried, and solubilized in 10 μl of methanol. We analyzed 1 μl of each sample by GC-MS, using the HP5 capillary column (30 m × 0.25 mm, 0.25 μm; Agilent). Helium was employed as carrier gas at a rate of 1.0 ml min^−1^. The temperature of the GC injector was maintained at 230°C; while the oven temperature was initially set at 40°C for 5 min, it was subsequently increased to 280°C for 5 min with an increment of 20°C/min, resulting in a total separation time of 20 min. The temperature of the analyzer was kept at 250°C. The collision energy was set to 70 eV, and the generated fragment ions were analyzed in a mass range of 20 to 450 *m/z*. The presence of arsenic compounds in the analyzed samples was confirmed by comparing the fragmentation spectra of the samples and the corresponding retention times with those of MMA and DMA standard solutions. Finally, the presence of MMAs and DMAs in the samples was confirmed by matching the experimental fragmentation spectra with those published in the NIST 05 mass spectral library. According to the NIST guidelines, the identification was reliable when the matching values were higher than 700 (37). The analyses of the samples were performed in technical triplicates.

### Co-IP assay.

The protein-protein interaction between *Tt*SmtB and *Tt*ArsM was *in vitro* verified via coimmunoprecipitation assays that employed recombinant *Tt*SmtB, recombinant His-tagged-*Tt*ArsM, anti-*Tt*SmtB antibodies (GeneCust), and His tag antibodies (Sigma-Aldrich). His tag removal from recombinant *Tt*SmtB was performed as previously described (22).

A typical co-IP mixture contained 1 ml of co-IP buffer (50 mM Tris-HCl, pH 7.5, 150 mM NaCl, 10% glycerol, and 0.1% Triton X-100), 5 μg of *Tt*SmtB, and 5 μg of His-tagged *Tt*ArsM and was incubated at 4°C for 2 h in continuous rotation. In some cases, arsenite, arsenate, cadmium, and antimony at 1:0, 1:25, 1:50, 1:100 molar ratios, respectively, preincubated with *Tt*SmtB for 10 min at 60°C, were added. As controls, *Tt*ArsM (5 μg), *Tt*SmtB (5 μg), and *Tt*GalA (40) were also separately incubated under the same conditions. All the samples were subjected to immunoprecipitation using 2 μl of purified anti-*Tt*SmtB antibodies (2 μg/μl) (GeneCust) for 3 h at 4°C in continuous rotation before adding 15 μl of protein A-Sepharose beads (Sigma-Aldrich) and allowing the incubation to continue for a further 16 h at 4°C. The formed immunocomplexes were washed with co-IP buffer and analyzed by Western blot on 15% SDS-PAGE, using polyvinylidene difluoride (PVDF) membranes (Millipore) and anti-polyhistidine-peroxidase antibodies (Sigma-Aldrich) diluted 1:10,000, as previously described (61).

ImageJ software (https://imagej.net/) was used for densitometric analysis of the formed bands, setting as maximum value the intensity of the band of the sample containing *Tt*ArsM and *Tt*SmtB in the absence of any metal. Each experiment was performed in three technical and two biological replicates; statistical analysis was performed using one-way analysis of variance (ANOVA), and significant differences are indicated as *, *P < *0.05; **, *P < *0.01; and ***, *P < *0.001.

### EMSAs.

Electrophoretic mobility shift assays (EMSAs) were performed to determine the *in vitro* binding of *Tt*SmtB to the promoter region upstream of *TtarsM*. The 108-bp chromosomal region that encompasses the start codon of the *TtarsM* gene and the 105-bp-long region upstream, denoted as *p_arsM_*, was PCR amplified with *Taq* DNA polymerase (Thermo Fisher Scientific) using the *p_arsM_ Fw* and *p_arsM_ Rv* primers (Table S3). EMSA reactions were set up as previously described (23, 62) in the presence of 1 μg of poly(dI-dC), 20 ng *p_arsM_*, and increasing concentrations of *Tt*SmtB (1, 2, 3, 5, and 10 μM, considering *Tt*SmtB as a dimer) using SYBR Gold nucleic acid gel stain for band detection. The EMSA reactions that simultaneously employed *Tt*SmtB and *Tt*ArsM were set up in identical conditions using 3 μM of each protein alone or in combination.

### Arsenic tolerance of E. coli expressing *Tt*ArsM.

The following strains were inoculated in 10 ml of LB precultures, supplemented with kanamycin (50 μg/ml) and chloramphenicol (33 μg/ml): (i) E. coli BL21-CodonPlus (DE3)-RIL, pET30*/TtarsM*; (ii) E. coli BL21-CodonPlus (DE3)-RIL, pET30*/TtarsM C77S*; (iii) E. coli BL21-CodonPlus (DE3)-RIL, pET30*/TtarsM H40A*; (iv) E. coli BL21-CodonPlus (DE3)-RIL, pET30*/TtarsM H179A*; and (v) E. coli BL21-CodonPlus (DE3)-RIL, pET30 (control). The precultures were incubated at 37°C for 16 h at 180 rpm. Subsequently, 50-ml LB cultures, supplemented with antibiotics, were inoculated with the precultures to initial OD_600_ of 0.08 and incubated at 37°C and 180 rpm until an OD_600_ of 0.6 was reached (exponential growth). At that point, protein expression was induced with 1 mM IPTG, and the cultures were incubated at 37°C and 180 rpm for 3 additional hours. From these growing cells, fresh LB cultures were inoculated to OD_600_ of 0.05 and distributed to 24-well plates (1 ml per well) containing LB medium supplemented with 1 mM IPTG, kanamycin (50 μg/ml), chloramphenicol (33 μg/ml), and increasing concentrations of arsenate and arsenite (from 2.5 mM to 7.0 mM). The MICs endpoint for each strain were determined as the lowest concentration of arsenite at which there was the difference between grown and start culture lower than 0.01 OD_600_ after 16 h of incubation at 37°C (22). All the cells up to the MIC value were able to grow if reinoculated in an arsenic-free medium. The reported values are the average of three biological replicates.

### ThermoCas9 editing and targeting constructs.

The plasmids used for the ThermoCas9-based targeting and editing experiments are listed in Table S4. The vector pMK18 was used as the template for the construction of the ThermoCas9-based targeting and editing plasmids and the employed primers, the DNA templates, and the DNA fragments, which are listed in Table S4. The *thermoCas9* gene was codon harmonized according to T. thermophilus HB27 codon usage using the Galaxy/Codon Harmonizer online tool (63), and it was synthesized (Twist Bioscience) (Table S5). The DNA fragments were designed with appropriate overhangs for NEBuilder HiFi DNA assembly (NEB), and they were obtained through PCR with Q5 polymerase (NEB). The PCR products were subjected to 1% agarose gel electrophoresis, and they were purified using a Zymogen gel DNA recovery kit (Zymo Research). The assembly reactions were transformed to chemically competent E. coli DH5α cells (NEB), and the cells were plated on LB agar plates supplemented with kanamycin (50 μg/ml). Single colonies were inoculated in LB medium supplemented with kanamycin (50 μg/ml) for overnight incubation at 37°C. Plasmid material was isolated using the GeneJet plasmid miniprep kit (Thermo Fisher Scientific) and sequence verified (GATC Biotech), and 300 ng of each plasmid (pMK-ThermoCas9-NT/sp1/sp2 and pMK-ThermoCas9-HR-NT/sp1/sp2) was transformed to either T. thermophilus HB27 cells (22), as indicated per experimental process.

10.1128/mBio.02813-21.4 TABLE S4 List of the PCR products used for the HiFi assembly reactions to construct the ThermoCas9-based targeting and editing plasmids. The primers and templates used for the PCR reactions are also included in this list. Download Table S4, DOCX file, 0.02 MB. Copyright © 2021 Gallo et al. 2021 Gallo et al. https://creativecommons.org/licenses/by/4.0/ This content is distributed under the terms of the Creative Commons Attribution 4.0 International license.

10.1128/mBio.02813-21.5 TABLE S5 Sequence of the synthesized *thermoCas9* gene, which is codon harmonized according to the codon usage of T. thermophilus HB27. Download Table S5, DOCX file, 0.01 MB. Copyright © 2021 Gallo et al. 2021 Gallo et al. https://creativecommons.org/licenses/by/4.0/ This content is distributed under the terms of the Creative Commons Attribution 4.0 International license.

The obtained plasmid, pMK-ThermoCas9-NT, was used as the backbone to construct a new plasmid to obtain the deletion of the *TtarsX* gene (Table S4) and the insertion of the gene coding sYFP (27). The obtained plasmid (pMK-ThermoCas9-HR-syfp) was used to transform T. thermophilus HB27 *ΔTtarsM* cells as already described to obtain the strain denoted Δ*TtarsM*-Δ*TtarsX* (*syfp*).

### Arsenic tolerance of T. thermophilus HB27 wild-type and mutant strains.

Exponentially growing precultures of T. thermophilus HB27 (control), T. thermophilus HB27 Δ*TtarsM*, and T. thermophilus HB27 Δ*TtarsM-*Δ*TtarsX* (*syfp*) were diluted to an OD_600_ of 0.08 in 10-ml TM cultures containing increasing concentrations of arsenite and arsenate (from 0.1 mM to 50 mM). The cultures were incubated aerobically at 70°C for 18 h, and the MIC values were determined as the lowest concentrations of arsenite and arsenate that completely inhibited the growth of a strain (22). The reported values are the average of three biological replicates.

### Bioreporter activity measurement.

Overnight cultures of T. thermophilus HB27 Δ*TtarsM*-Δ*TtarsX* (*syfp*) strain were diluted to an OD_600_ of 0.08 in TM medium and then grown aerobically at 70°C until an OD_600_ of 0.5 was reached. The cultures were divided into samples of 5 ml each and subsequently supplemented with increasing concentrations of arsenite and arsenate (0.5 μM to 4 μM). After 1 h of incubation at 70°C, 200 μl of each cell sample were removed and centrifuged for 5 min at 6,000 rpm. The pellets were washed twice with equal volumes of phosphate-buffered saline (PBS) 1× and resuspended with equal volumes of PBS 1× before being distributed into a 96-well plate. sYFP fluorescence intensity of each sample was measured employing a Synergy HTX multimode microplate reader (BioTek), using excitation and emission wavelengths of 458 nm and 540 nm, respectively. The measured fluorescence intensities were normalized for the optical density of each sample at 600 nm. The measured fluorescence was reported as fluorescence relative expression, assuming that the fluorescence value of not-treated cells (control) was 1.

Each experiment was performed in three technical and biological replicates. Statistical analysis was performed using one-way ANOVA; significant differences are indicated as *, *P < *0.05; **, *P < *0.01; and ***, *P < *0.001.

## References

[1] Mukhopadhyay R, Rosen BP, Phung LT, Silver S. 2002. Microbial arsenic: from geocycles to genes and enzymes. FEMS Microbiol Rev 26:311–325. doi:10.1111/j.1574-6976.2002.tb00617.x. 12165430

[2] Chen Z, Wang Y, Jiang X, Fu D, Xia D, Wang H, Dong G, Li Q. 2017. Dual roles of AQDS as electron shuttles for microbes and dissolved organic matter involved in arsenic and iron mobilization in the arsenic-rich sediment. Sci Total Environ 574:1684–1694. doi:10.1016/j.scitotenv.2016.09.006. 27616712

[3] Yang H-C, Rosen BP. 2016. New mechanisms of bacterial arsenic resistance. Biomed J 39:5–13. doi:10.1016/j.bj.2015.08.003. 27105594 PMC6138428

[4] Gallo G, Puopolo R, Carbonaro M, Maresca E, Fiorentino G. 2021. Extremophiles, a nifty tool to face environmental pollution: from exploitation of metabolism to genome engineering. Int J Environ Res Public Health 18:5228. doi:10.3390/ijerph18105228. 34069056 PMC8157027

[5] Politi J, Spadavecchia J, Fiorentino G, Antonucci I, Casale S, De Stefano L. 2015. Interaction of Thermus thermophilus ArsC enzyme and gold nanoparticles naked-eye assays speciation between As(III) and As(V). Nanotechnology 26:435703. doi:10.1088/0957-4484/26/43/435703. 26436536

[6] Politi J, Spadavecchia J, Fiorentino G, Antonucci I, De Stefano L. 2016. Arsenate reductase from *Thermus thermophilus* conjugated to polyethylene glycol-stabilized gold nanospheres allow trace sensing and speciation of arsenic ions. J R Soc Interface 13:20160629. doi:10.1098/rsif.2016.0629. 27707908 PMC5095221

[7] Rosen BP. 2002. Biochemistry of arsenic detoxification. FEBS Lett 529:86–92. doi:10.1016/s0014-5793(02)03186-1. 12354618

[8] Liu Z, Rensing C, Rosen BP. 2011. Toxicity: resistance pathways for metalloids and toxic metals, p 1–13. *In* Scott RA (ed), Encyclopedia of inorganic and bioinorganic chemistry. John Wiley & Sons, Ltd., Hoboken, NJ.

[9] Aulitto M, Gallo G, Puopolo R, Mormone A, Limauro D, Contursi P, Piochi M, Bartolucci S, Fiorentino G. 2021. Genomic insight of Alicyclobacillus mali FL18 isolated from an arsenic-rich hot spring. Front Microbiol 12:639697. doi:10.3389/fmicb.2021.639697. 33897644 PMC8060452

[10] Mukhopadhyay R, Rosen BP. 2002. Arsenate reductases in prokaryotes and eukaryotes. Environ Health Perspect 110:745–748. doi:10.1289/ehp.02110s5745. 12426124 PMC1241237

[11] Chen C-M, Misra TK, Silver S, Rosen BP. 1986. Nucleotide sequence of the structural genes for an anion pump. The plasmid-encoded arsenical resistance operon. J Biol Chem 261:15030–15038. doi:10.1016/S0021-9258(18)66824-3. 3021763

[12] Wu J, Rosen BP. 1991. The ArsR protein is a trans‐acting regulatory protein. Mol Microbiol 5:1331–1336. doi:10.1111/j.1365-2958.1991.tb00779.x. 1838573

[13] Qin J, Rosen BP, Zhang Y, Wang G, Franke S, Rensing C. 2006. Arsenic detoxification and evolution of trimethylarsine gas by a microbial arsenite S-adenosylmethionine methyltransferase. Proc Natl Acad Sci USA 103:2075–2080. doi:10.1073/pnas.0506836103. 16452170 PMC1413689

[14] Chen J, Madegowda M, Bhattacharjee H, Rosen BP. 2015. ArsP: a methylarsenite efflux permease. Mol Microbiol 98:625–635. doi:10.1111/mmi.13145. 26234817 PMC4681449

[15] Chen S-C, Sun G-X, Yan Y, Konstantinidis KT, Zhang S-Y, Deng Y, Li X-M, Cui H-L, Musat F, Popp D, Rosen BP, Zhu Y-G. 2020. The great oxidation event expanded the genetic repertoire of arsenic metabolism and cycling. Proc Natl Acad Sci USA 117:10414–10421. doi:10.1073/pnas.2001063117. 32350143 PMC7229686

[16] Chen S-C, Sun G-X, Rosen BP, Zhang S-Y, Deng Y, Zhu B-K, Rensing C, Zhu Y-G. 2017. Recurrent horizontal transfer of arsenite methyltransferase genes facilitated adaptation of life to arsenic. Sci Rep 7:1–11. doi:10.1038/s41598-017-08313-2. 28798375 PMC5552862

[17] Chen J, Rosen BP. 2020. The arsenic methylation cycle: how microbial communities adapted methylarsenicals for use as weapons in the continuing war for dominance. Front Environ Sci 8. doi:10.3389/fenvs.2020.00043.

[18] Gihring TM, Druschel GK, McCleskey RB, Hamers RJ, Banfield JF. 2001. Rapid arsenite oxidation by Thermus aquaticus and Thermus thermophilus: field and laboratory investigations. Environ Sci Technol 35:3857–3862. doi:10.1021/es010816f. 11642444

[19] Saltikov CW, Olson BH. 2002. Homology of Escherichia coli R773 arsA, arsB, and arsC genes in arsenic-resistant bacteria isolated from raw sewage and arsenic-enriched creek waters. Appl Environ Microbiol 68:280–288. doi:10.1128/AEM.68.1.280-288.2002. 11772637 PMC126541

[20] Qin J, Lehr CR, Yuan C, Le XC, McDermott TR, Rosen BP. 2009. Biotransformation of arsenic by a Yellowstone thermoacidophilic eukaryotic alga. Proc Natl Acad Sci USA 106:5213–5217. doi:10.1073/pnas.0900238106. 19276121 PMC2664070

[21] Henne A, Brüggemann H, Raasch C, Wiezer A, Hartsch T, Liesegang H, Johann A, Lienard T, Gohl O, Martinez-Arias R, Jacobi C, Starkuviene V, Schlenczeck S, Dencker S, Huber R, Klenk HP, Kramer W, Merkl R, Gottschalk G, Fritz HJ. 2004. The genome sequence of the extreme thermophile Thermus thermophilus. Nat Biotechnol 22:547–553. doi:10.1038/nbt956. 15064768

[22] Antonucci I, Gallo G, Limauro D, Contursi P, Ribeiro AL, Blesa A, Berenguer J, Bartolucci S, Fiorentino G. 2017. An ArsR/SmtB family member regulates arsenic resistance genes unusually arranged in Thermus thermophilus HB27. Microb Biotechnol 10:1690–1701. doi:10.1111/1751-7915.12761. 28696001 PMC5658604

[23] Antonucci I, Gallo G, Limauro D, Contursi P, Ribeiro AL, Blesa A, Berenguer J, Bartolucci S, Fiorentino G. 2018. Characterization of a promiscuous cadmium and arsenic resistance mechanism in Thermus thermophilus HB27 and potential application of a novel bioreporter system. Microb Cell Fact 17:78. doi:10.1186/s12934-018-0918-7. 29776370 PMC5960188

[24] Del Giudice I, Limauro D, Pedone E, Bartolucci S, Fiorentino G. 2013. A novel arsenate reductase from the bacterium Thermus thermophilus HB27: its role in arsenic detoxification. Biochim Biophys Acta 1834:2071–2079. doi:10.1016/j.bbapap.2013.06.007. 23800470

[25] Puopolo R, Sorrentino I, Gallo G, Piscitelli A, Giardina P, Le Goff A, Fiorentino G. 2021. Self-assembling thermostable chimeras as new platform for arsenic biosensing. Sci Rep 11:2991. doi:10.1038/s41598-021-82648-9. 33542380 PMC7862302

[26] Busenlehner LS, Pennella MA, Giedroc DP. 2003. The SmtB/ArsR family of metalloregulatory transcriptional repressors: structural insights into prokaryotic metal resistance. FEMS Microbiol Rev 27:131–143. doi:10.1016/S0168-6445(03)00054-8. 12829264

[27] Blesa A, Berenguer J. 2015. Contribution of vesicle-protected extracellular DNA to horizontal gene transfer in Thermus spp. Int Microbiol 18:177–187. doi:10.2436/20.1501.01.248. 27036745

[28] Carpentieri A, Gamberi T, Modesti A, Amoresano A, Colombini B, Nocella M, Bagni MA, Fiaschi T, Barolo L, Gulisano M, Magherini F. 2016. Profiling carbonylated proteins in heart and skeletal muscle mitochondria from trained and untrained mice. J Proteome Res 15:3666–3678. doi:10.1021/acs.jproteome.6b00475. 27571187

[29] Rao ST, Rossmann MG. 1973. Comparison of super-secondary structures in proteins. J Mol Biol 76:241–256. doi:10.1016/0022-2836(73)90388-4. 4737475

[30] Schubert HL, Blumenthal RM, Cheng X. 2003. Many paths to methyltransfer: a chronicle of convergence. Trends Biochem Sci 28:329–335. doi:10.1016/S0968-0004(03)00090-2. 12826405 PMC2758044

[31] Huang K, Xu Y, Packianathan C, Gao F, Chen C, Zhang J, Shen Q, Rosen BP, Zhao F. 2018. Arsenic methylation by a novel ArsM As (III) S‐adenosylmethionine methyltransferase that requires only two conserved cysteine residues. Mol Microbiol 107:265–276. doi:10.1111/mmi.13882. 29134708 PMC5760297

[32] Gallo G, Antonucci I, Pirone L, Amoresano A, Contursi P, Limauro D, Pedone E, Bartolucci S, Fiorentino G. 2019. A physicochemical investigation on the metal binding properties of TtSmtB, a thermophilic member of the ArsR/SmtB transcription factor family. Int J Biol Macromol 138:1056–1063. doi:10.1016/j.ijbiomac.2019.07.174. 31356933

[33] Singh J, Vijay S, Mansuri R, Rawal R, Kadian K, Sahoo GC, Kumar M, Sharma A. 2019. Computational and experimental elucidation of Plasmodium falciparum phosphoethanolamine methyltransferase inhibitors: pivotal drug target. PLoS One 14:e0221032. doi:10.1371/journal.pone.0221032. 31437171 PMC6705855

[34] Patil NA, Basu B, Deobagkar DD, Apte SK, Deobagkar DN. 2017. Putative DNA modification methylase DR_C0020 of Deinococcus radiodurans is an atypical SAM dependent C-5 cytosine DNA methylase. Biochim Biophys Acta Gen Subj 1861:593–602. doi:10.1016/j.bbagen.2016.12.025. 28038990

[35] Dou L, Yan F, Pang J, Zheng D, Li D, Gao L, Wang L, Xu Y, Shi J, Wang Q, Zhou L, Shen N, Singh P, Wang L, Li Y, Gao Y, Liu T, Chen C, Al-Kali A, Litzow MR, Chi Y-I, Bode AM, Liu C, Huang H, Liu D, Marcucci G, Liu S, Yu L. 2019. Protein lysine 43 methylation by EZH1 promotes AML1-ETO transcriptional repression in leukemia. Nat Commun 10:1–15. doi:10.1038/s41467-019-12960-6. 31699991 PMC6838331

[36] Huang M, Ting Wang Y, Ho PC. 2008. Quantification of arsenic compounds using derivatization, solvent extraction and liquid chromatography electrospray ionization tandem mass spectrometry. J Pharm Biomed Anal 48:1381–1391. doi:10.1016/j.jpba.2008.09.018. 18977105

[37] Stein SE. 1994. Estimating probabilities of correct identification from results of mass spectral library searches. J Am Soc Mass Spectrom 5:316–323. doi:10.1016/1044-0305(94)85022-4. 24222569

[38] Zhang J, Cao T, Tang Z, Shen Q, Rosen BP, Zhao F-J. 2015. Arsenic methylation and volatilization by arsenite S-adenosylmethionine methyltransferase in Pseudomonas alcaligenes NBRC14159. Appl Environ Microbiol 81:2852–2860. doi:10.1128/AEM.03804-14. 25681184 PMC4375323

[39] Wang P-P, Sun G-X, Zhu Y-G. 2014. Identification and characterization of arsenite methyltransferase from an archaeon, Methanosarcina acetivorans C2A. Environ Sci Technol 48:12706–12713. doi:10.1021/es503869k. 25295694

[40] Aulitto M, Fusco S, Fiorentino G, Limauro D, Pedone E, Bartolucci S, Contursi P. 2017. Thermus thermophilus as source of thermozymes for biotechnological applications: homologous expression and biochemical characterization of an α-galactosidase. Microb Cell Fact 16:1–10. doi:10.1186/s12934-017-0638-4. 28193276 PMC5307791

[41] Osman D, Cavet JS. 2010. Bacterial metal-sensing proteins exemplified by ArsR–SmtB family repressors. Nat Prod Rep 27:668–680. doi:10.1039/b906682a. 20442958

[42] Tanous C, Soutourina O, Raynal B, Hullo M-F, Mervelet P, Gilles A-M, Noirot P, Danchin A, England P, Martin-Verstraete I. 2008. The CymR regulator in complex with the enzyme CysK controls cysteine metabolism in Bacillus subtilis. J Biol Chem 283:35551–35560. doi:10.1074/jbc.M805951200. 18974048

[43] Fisher SH, Wray LV. 2008. Bacillus subtilis glutamine synthetase regulates its own synthesis by acting as a chaperone to stabilize GlnR-DNA complexes. Proc Natl Acad Sci USA 105:1014–1019. doi:10.1073/pnas.0709949105. 18195355 PMC2242682

[44] Cava F, Laptenko O, Borukhov S, Chahlafi Z, Blas‐Galindo E, Gómez‐Puertas P, Berenguer J. 2007. Control of the respiratory metabolism of Thermus thermophilus by the nitrate respiration conjugative element NCE. Mol Microbiol 64:630–646. doi:10.1111/j.1365-2958.2007.05687.x. 17462013

[45] Mougiakos I, Mohanraju P, Bosma EF, Vrouwe V, Bou MF, Naduthodi MIS, Gussak A, Brinkman RBL, Van Kranenburg R, Van Der Oost J. 2017. Characterizing a thermostable Cas9 for bacterial genome editing and silencing. Nat Commun 8:1–11. doi:10.1038/s41467-017-01591-4. 29162801 PMC5698299

[46] Blesa A, Baquedano I, Quintáns NG, Mata CP, Castón JR, Berenguer J. 2017. The transjugation machinery of Thermus thermophilus: identification of TdtA, an ATPase involved in DNA donation. PLoS Genet 13:e1006669. doi:10.1371/journal.pgen.1006669. 28282376 PMC5365140

[47] Roy R, Samanta S, Patra S, Mahato NK, Saha RP. 2018. In silico identification and characterization of sensory motifs in the transcriptional regulators of the ArsR-SmtB family. Metallomics 10:1476–1500. doi:10.1039/c8mt00082d. 30191942

[48] Ajees AA, Marapakala K, Packianathan C, Sankaran B, Rosen BP. 2012. Structure of an As (III) S-adenosylmethionine methyltransferase: insights into the mechanism of arsenic biotransformation. Biochemistry 51:5476–5485. doi:10.1021/bi3004632. 22712827 PMC3447999

[49] Dheeman DS, Packianathan C, Pillai JK, Rosen BP. 2014. Pathway of human AS3MT arsenic methylation. Chem Res Toxicol 27:1979–1989. doi:10.1021/tx500313k. 25325836 PMC4237493

[50] Fusco S, Aulitto M, Iacobucci I, Crocamo G, Pucci P, Bartolucci S, Monti M, Contursi P. 2020. The interaction between the F55 virus-encoded transcription regulator and the RadA host recombinase reveals a common strategy in Archaea and Bacteria to sense the UV-induced damage to the host DNA. Biochim Biophys Acta Gene Regul Mech 1863:194493. doi:10.1016/j.bbagrm.2020.194493. 32014611

[51] Le Y, Fu Y, Sun J. 2020. Genome editing of the anaerobic thermophile Thermoanaerobacter ethanolicus using thermostable Cas9. Appl Environ Microbiol 87:e01773-20. doi:10.1128/AEM.01773-20. 33067194 PMC7755235

[52] Adalsteinsson BT, Kristjansdottir T, Merre W, Helleux A, Dusaucy J, Tourigny M, Fridjonsson O, Hreggvidsson GO. 2021. Efficient genome editing of an extreme thermophile, Thermus thermophilus, using a thermostable Cas9 variant. Sci Rep 11:1–15. doi:10.1038/s41598-021-89029-2. 33953310 PMC8100143

[53] Monti M, Orru S, Pagnozzi D, Pucci P. 2005. Functional proteomics. Clin Chim Acta 357:140–150. doi:10.1016/j.cccn.2005.03.019. 15946657

[54] Kumar S, Stecher G, Li M, Knyaz C, Tamura K. 2018. MEGA X: molecular evolutionary genetics analysis across computing platforms. Mol Biol Evol 35:1547–1549. doi:10.1093/molbev/msy096. 29722887 PMC5967553

[55] Wang P-P, Bao P, Sun G-X. 2015. Identification and catalytic residues of the arsenite methyltransferase from a sulfate-reducing bacterium, Clostridium sp. BXM. FEMS Microbiol Lett 362:1–8. doi:10.1093/femsle/fnu003. 25790486

[56] Wang G, Kennedy SP, Fasiludeen S, Rensing C, DasSarma S. 2004. Arsenic resistance in Halobacterium sp. strain NRC-1 examined by using an improved gene knockout system. J Bacteriol 186:3187–3194. doi:10.1128/JB.186.10.3187-3194.2004. 15126481 PMC400623

[57] Sievers F, Wilm A, Dineen D, Gibson TJ, Karplus K, Li W, Lopez R, McWilliam H, Remmert M, Söding J, Thompson JD, Higgins DG. 2011. Fast, scalable generation of high‐quality protein multiple sequence alignments using Clustal Omega. Mol Syst Biol 7:539. doi:10.1038/msb.2011.75. 21988835 PMC3261699

[58] Zhang Y. 2008. I-TASSER server for protein 3D structure prediction. BMC Bioinformatics 9:40. doi:10.1186/1471-2105-9-40. 18215316 PMC2245901

[59] HeeShin W. 2014. Prediction of protein structure and interaction by GALAXY protein modeling programs. Biodesign 2:1–11.

[60] Macindoe G, Mavridis L, Venkatraman V, Devignes M-D, Ritchie DW. 2010. HexServer: an FFT-based protein docking server powered by graphics processors. Nucleic Acids Res 38:W445–W449. doi:10.1093/nar/gkq311. 20444869 PMC2896144

[61] Fiorentino G, Ronca R, Cannio R, Rossi M, Bartolucci S. 2007. MarR-like transcriptional regulator involved in detoxification of aromatic compounds in Sulfolobus solfataricus. J Bacteriol 189:7351–7360. doi:10.1128/JB.00885-07. 17675388 PMC2168448

[62] Fiorentino G, Del Giudice I, Bartolucci S, Durante L, Martino L, Del Vecchio P. 2011. Identification and physicochemical characterization of BldR2 from Sulfolobus solfataricus, a novel archaeal member of the MarR transcription factor family. Biochemistry 50:6607–6621. doi:10.1021/bi200187j. 21714562

[63] Claassens NJ, Siliakus MF, Spaans SK, Creutzburg SCA, Nijsse B, Schaap PJ, Quax TEF, Van Der Oost J. 2017. Improving heterologous membrane protein production in Escherichia coli by combining transcriptional tuning and codon usage algorithms. PLoS One 12:e0184355. doi:10.1371/journal.pone.0184355. 28902855 PMC5597330

