# Supplementary material for: A Hyperthermoactive-Cas9 Editing Tool Reveals the Role of a Unique Arsenite Methyltransferase in the Arsenic Resistance System of Thermus thermophilus HB27
Source: mBio. 2021 Dec 7;12(6):e02813-21. doi: 10.1128/mBio.02813-21 (PMC8649762; doi:10.1128/mBio.02813-21)
Supplement: TABLE S1 [file mbio.02813-21-st001.docx]

**TABLE S1.**

| **Protein name** | **Accession number** | ***T. therm.* extract** | | |
| --- | --- | --- | --- | --- |
|  |  | **NT** | **AsV** | **AsIII** |
| Pyruvate dehydrogenase E1 component | Q72GP7 |  | X | X |
| Pyrroline-5-carboxylate reductase (proC) | P54893 |  |  | X |
| Hypothetical conserved protein(Resitric-modif protein Tth111, Q49LI5) | Q72GH1 |  |  | X |
| Homocitrate synthase(lys20) | O87198 | X | X | X |
| Hypothetical conserved protein  (Thymidylate kinase, F6DGR2) | Q72JZ6 |  | x | X |
| Acetolactate synthase | Q5SJ01 | X |  |  |
| 2-isopropylmalate (leuA)14 | Q72JC9 | X |  |  |
| Transketolase | Q5SM35 | X |  |  |
| Uncharacterized protein | Q5SJM2 | X |  |  |
| Chaperone protein DnaK(Hsp70) | Q72IK5 | X |  | X |
| Copper-exporting ATPase | Q72HW1 | X |  |  |
| Nicotinate phosphoribosyltransferase | Q72L13 | X | X | X |
| ABC transporter ATP-binding protein | Q72HJ9 |  |  | X |
| Phosphoenolpyruvate carboxykinase [ATP] (pckA) | Q72GY7 |  |  | X |
| 2-Phosphoglycerate kinase | Q5SKZ8 | X |  |  |
| (Neo) pullulanase | Q5SI17 | X | X |  |
| PDH Dihydrolipoamide acetyltransferase | Q5SLV9 | X | X |  |
| AcetylCoA Biotin carboxylase | Q5SJ91 | X | X |  |
| 60kDa chaperonin (groL) P614905 | P61490 | X | X |  |
| Aspartate-tRNA (Asp/Asn) ligase (aspS2) | Q5SIC2 | X | X | X |
| Phosphoglycerate kinase | Q72LD8 |  | X |  |
| Acetolatate synthase | Q72JC6 |  | X |  |
| Dihydrolipoamide acetyltransferase | Q72GP6 |  |  | X |
| Dihydrolipoyl dehydrogenase | Q72GU5 |  |  | X |
| Aspartate-tRNA (Asp/Asn) ligase (aspS2) | Q72IP5 | X | X | X |
| Pyridoxal 5'-phosphate synthase sub PdxS (pdxS) | Q72KG1 | X |  | X |
| Putative dehydratase | Q72IR3 | X |  | X |
| Hydrolase (HAD superfamily) | Q72GG4 |  |  | X |
| 4-OH-3-MeBut-2-en-1-yl diP synthase (ispG) | Q5SLI8 | X |  |  |
| Ribonuclease | Q5SLP1 | X |  |  |
| Heat shock protein (hslU) | Q5SKL3 | X | X |  |
| Hypothetical conserved protein | Q746C0 | X | X | X |
| Elongation factor Tu-B (tufB) | P60339 | X | X |  |
| FAD/FMN-containing dehydrogenase | Q5SMA3 |  | X |  |
| Precorrin-6Y C5, 15-methyltransferase | Q746P3 |  | X | X |
| Riboflavin biosynthesis protein RibBA (ribA) | Q72JS1 |  | X | X |
| Iron-sulfur cluster-binding protein | Q72IY0 |  | X | X |
| Cell division protein ftsA (ftsA) | Q72JP5 |  |  | X |
| tRNA (cytidine/uridine-2'-O-)-methyltransferase (trmJ) | Q72JF4 | X |  | X |
| Two-component response regulator (hslU) | Q72JY5 |  |  | X |
| Acyl carrier protein (acpP) | Q72LL3 | X | X | X |
| Glycerol-3-phosphate DH [NAD (P)+] (gpsA) | P61747 | X |  |  |
| Zinc-binding dehydrogenase | Q5SL93 |  | X |  |
| Hypothetical conserved protein | Q72LF0 |  | X | X |
| Histidine biosynthesis bifunctional P (HisIE) | P62350 | X | X |  |
| Uroporphyrin-III  C-methyltransferase | Q746N6 | X | X |  |
| Uracil phosphoribosyltransferase | Q72J35 |  | X |  |
| 30S ribosomal protein S3 (rpsC) | P62663 | X | X |  |
| Hypothetical membrane spanning protein | Q72L74 | X |  |  |
