# Supplementary figures and images for: A Hyperthermoactive-Cas9 Editing Tool Reveals the Role of a Unique Arsenite Methyltransferase in the Arsenic Resistance System of Thermus thermophilus HB27
Source: mBio. 2021 Dec 7;12(6):e02813-21. doi: 10.1128/mBio.02813-21 (PMC8649762; doi:10.1128/mBio.02813-21)

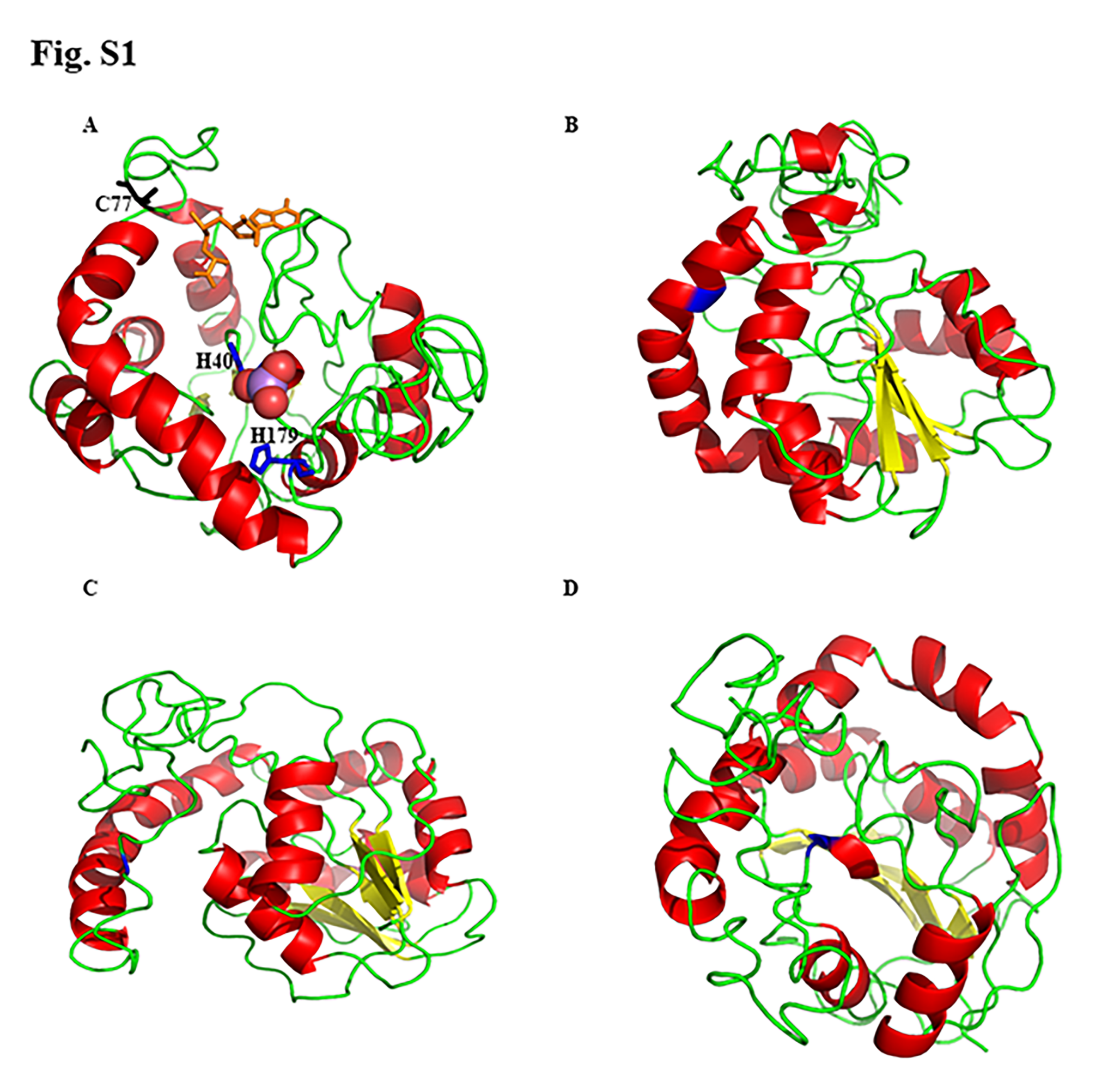

Supplement: FIG S1 [file mbio.02813-21-sf001.tif]

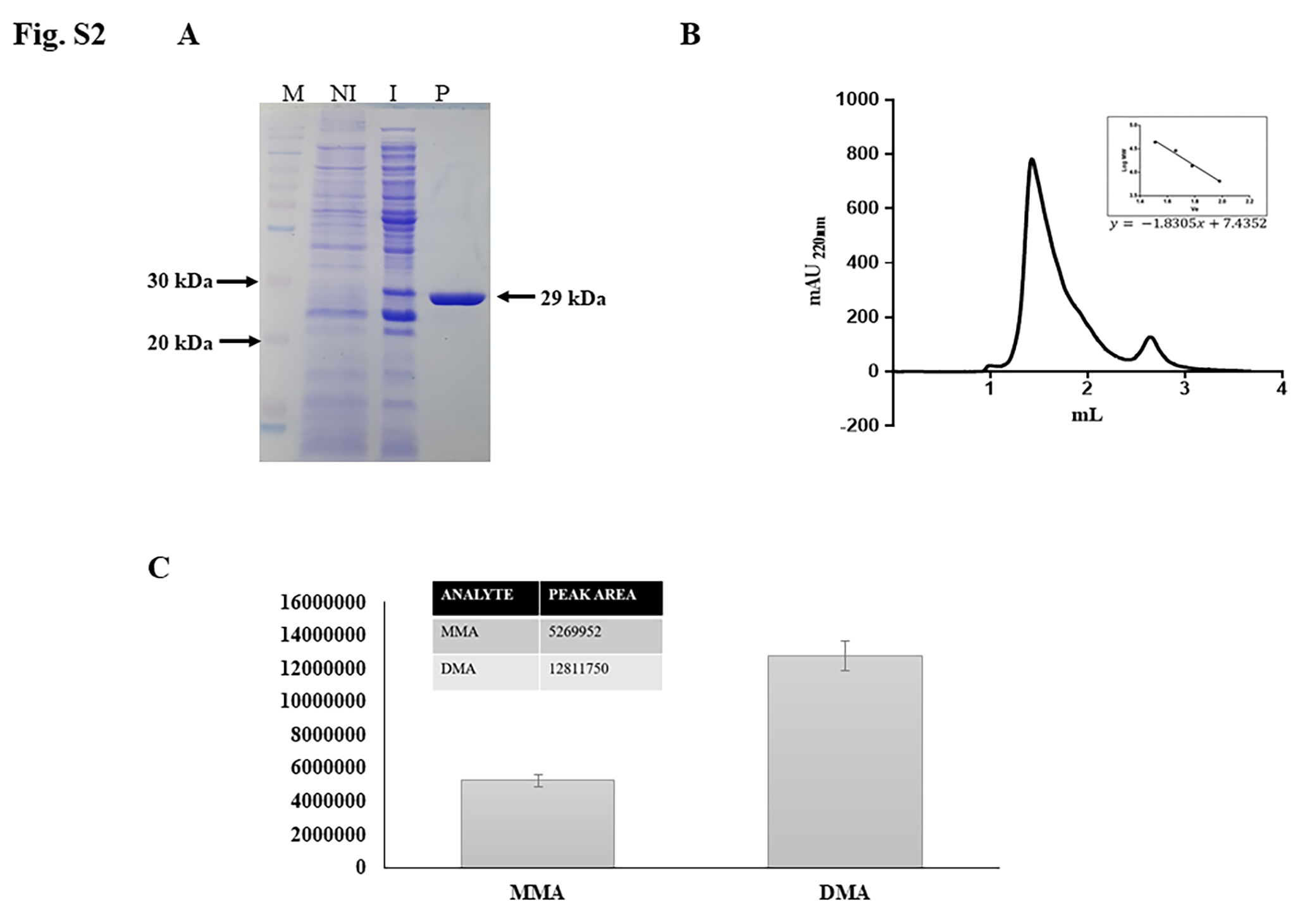

Supplement: FIG S2 [file mbio.02813-21-sf002.tif]

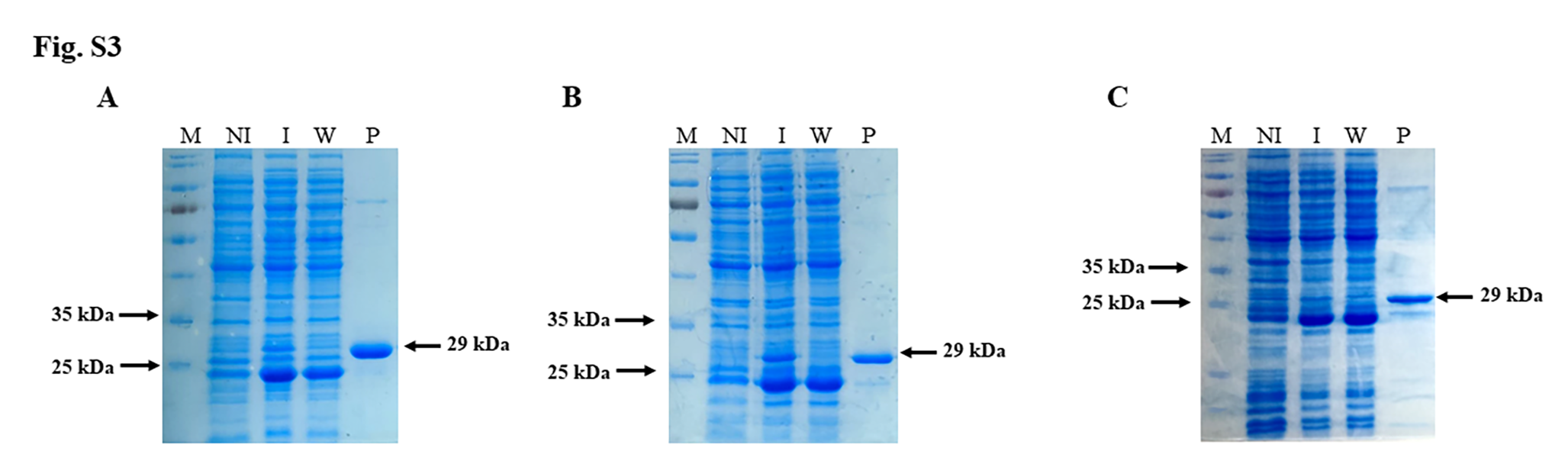

Supplement: FIG S3 [file mbio.02813-21-sf003.tif]

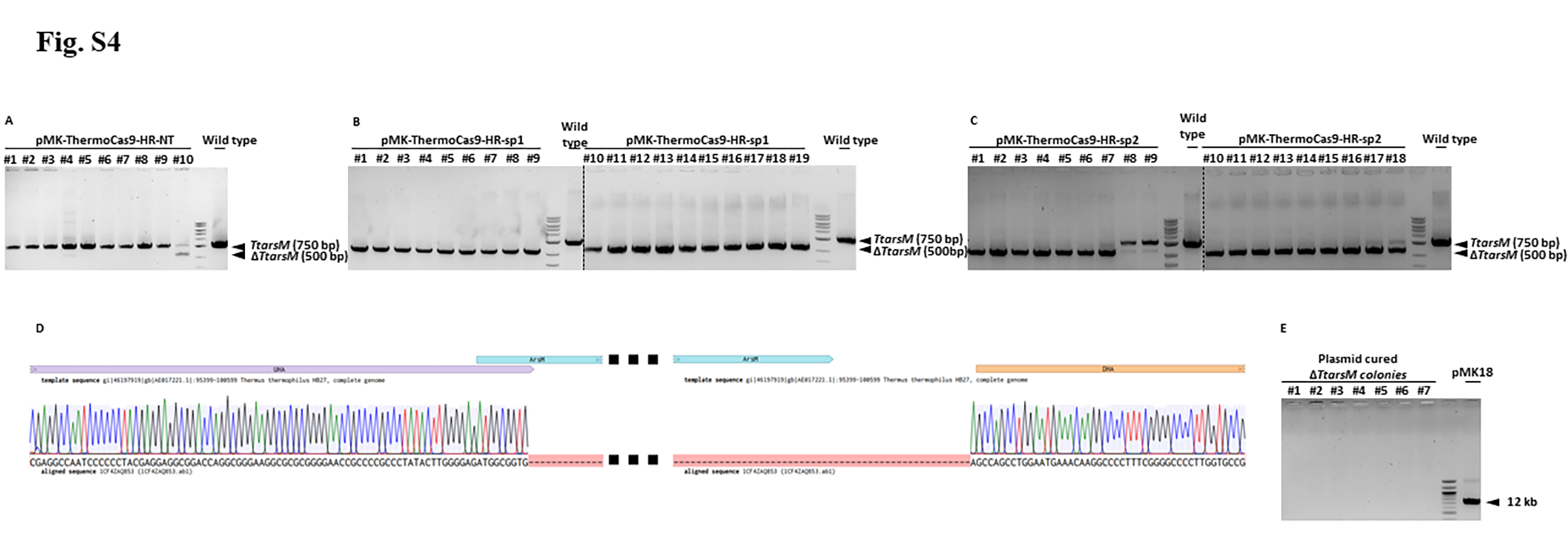

Supplement: FIG S4 [file mbio.02813-21-sf004.tif]

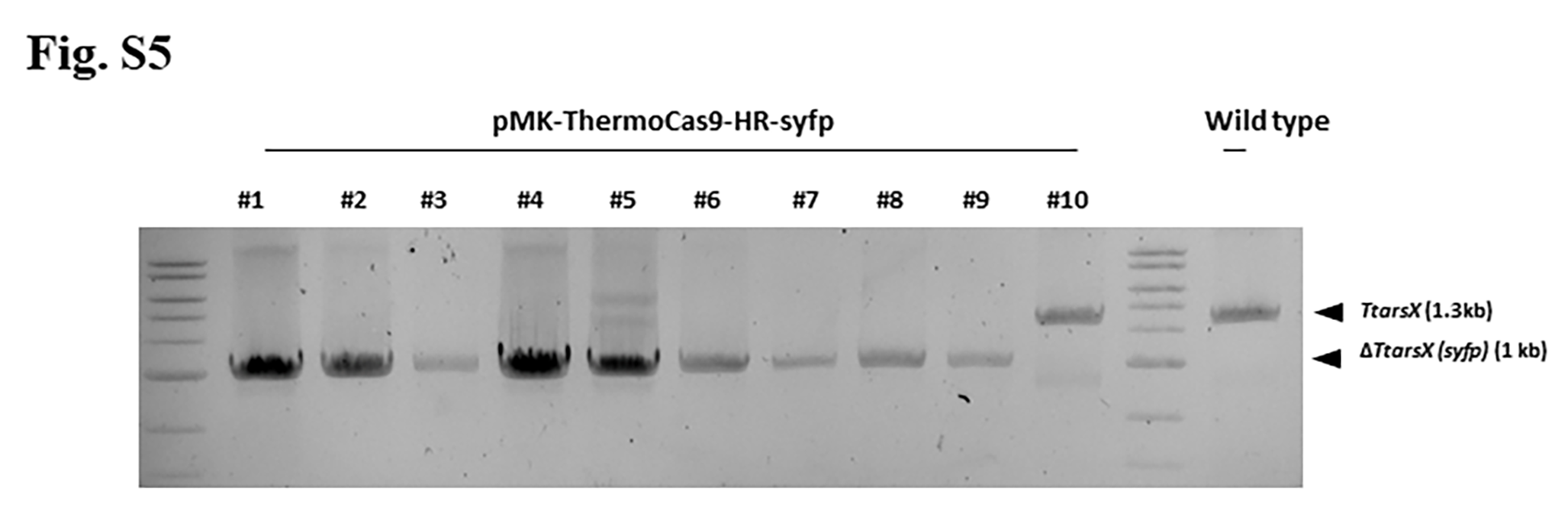

Supplement: FIG S5 [file mbio.02813-21-sf005.tif]
