# Supplementary material for: A Hyperthermoactive-Cas9 Editing Tool Reveals the Role of a Unique Arsenite Methyltransferase in the Arsenic Resistance System of Thermus thermophilus HB27
Source: mBio. 2021 Dec 7;12(6):e02813-21. doi: 10.1128/mBio.02813-21 (PMC8649762; doi:10.1128/mBio.02813-21)
Supplement: TABLE S2 [file mbio.02813-21-st002.docx]

**TABLE S2.**

| **Plasmid** | **Recovery temperature** | **Plating temperature** | **Number of formed colonies** | **Numbers of screened colonies** | | | |
| --- | --- | --- | --- | --- | --- | --- | --- |
|  |  |  |  | **Total** | **∆*Ttars*M** | **Wild type** | **Mixed** |
| **pMK- ThermoCas9-HR-NT** | 70˚C | 60˚C | 221 | 10 | 0 | 8 | 2 |
| **pMK- ThermoCas9-HR-sp1** | 70˚C | 60˚C | 79 | 19 | 19 | 0 | 0 |
| **pMK- ThermoCas9-HR-sp2** | 70˚C | 60˚C | 62 | 18 | 13 | 0 | 5 |
| **pMK- ThermoCas9- HR-sp1** | 70˚C | 65˚C | 3 | 3 | 3 | 0 | 0 |
| **pMK- ThermoCas9- HR-sp2** | 70˚C | 65˚C | 5 | 5 | 5 | 0 | 0 |
