# Supplementary material for: A Hyperthermoactive-Cas9 Editing Tool Reveals the Role of a Unique Arsenite Methyltransferase in the Arsenic Resistance System of Thermus thermophilus HB27
Source: mBio. 2021 Dec 7;12(6):e02813-21. doi: 10.1128/mBio.02813-21 (PMC8649762; doi:10.1128/mBio.02813-21)
Supplement: TABLE S3 [file mbio.02813-21-st003.docx]

**TABLE S3.**

| **Primer** | **Sequence** | **Primer function** |
| --- | --- | --- |
| *arsMfw* | AAACATATGGCGGTGAAGGCC | Forward primer for the amplification of the *TtarsM* gene from the *T. thermophilus* HB27 genome |
| *arsMrv* | AAAAAGCTTTCCCTTGTCCCAAA | Reverse primer for the amplification of the *TtarsM* gene from the *T. thermophilus* HB27 genome |
| H40Afw | CGGAAACCTCCGAGGCGGTGGGGAAGACGC | Forward primer for the contruction of the *TtarsM*  H40A mutant gene |
| H40Arv | GCGTCTTCCCCACCGCCTCGGAGGTTTCCG | Reverse primer for the contruction of the *TtarsM*  H40A mutant gene |
| C77Sfw | CCCCTGCCTCGCTGGCGTTGGTAAG | Forward primer for the contruction of the *TtarsM*  C77S mutant gene |
| C77Srv | CTTACCAACGCCAGCGAGGCAGGGG | Reverse primer for the contruction of the *TtarsM*  C77S mutant gene |
| H179Afw | AACGGGGTCCAGGGCGTGGAGGGCGAGG | Forward primer for the contruction of the *TtarsM*  H179A mutant gene |
| H179Arv | CCTCGCCCTCCACGCCCTGGACCCCGTT | Reverse primer for the contruction of the *TtarsM*  H179A mutant gene |
| *p_arsM_*Fw | GGAAGGGGAAGCGGTGTTT | Forward primer for the amplification of the *TtarsM* promoter region from the *T. thermophilus* HB27 genome |
| *p_arsM_*Rv | CGCCCTATACTTGGGGAGATG | Reverse primer for the amplification of the *TtarsM* promoter region from the *T. thermophilus* HB27 genome |
| BG15528 | AAGCCAGGGTTCCACGGTGGAAAAGGTGCTCCGGGTGGAAGAC | Primers used for the production of the amplicons that were used for the construction of the ThermoCas9-based targeting and editing vectors. For details see Table S4. |
| BG15529 | GGAGGCGGTCTTCCACCCGGAGCACCTTTTCCACCGTGGAACCC |  |
| BG15580 | GGGCCTTGTTTCATTCCAGGCTGGCTCACCGCCATCTCCCCAA |  |
| BG15581 | CCTATACTTGGGGAGATGGCGGTGAGCCAGCCTGGAATGAAAC |  |
| BG15582 | GGGGAGGTCTTCAGGTCCACCACCATTTTGGTCCAGGGGAACAT |  |
| BG15583 | GGGTTGATGTTCCCCTGGACCAAAATGGTGGTGGACCTGAAGA |  |
| BG15621 | TGTCCAGGCCGATCTTGTACTTCATATGCCCCTCCTTTCGTG |  |
| BG15622 | ATGAAGTACAAGATCGGCCTG |  |
| BG15625 | TCACAGGGGCCTGATCG |  |
| BG15626 | CGGCGAGACGATCAGGCCCCTGTGATGAGATCCGGCTGCTAACAA |  |
| BG15665 | TCATGTAACTCGCCTTGATCGTTGGTTGGGGTAGTCCAGCAC |  |
| BG15666 | GAGGGCCCACATCACCAACGCCCTCAACGCCCTTAACGGG |  |
| BG15667 | GCCTTACCGGGAGGAGGAGGTGGTCAACGCCCTTAACGGG |  |
| BG15669 | CCATGGGGTTACTGCGGATCTAGTCAACGCCCTTAACGGG |  |
| BG15670 | CTAGATCCGCAGTAACCCCATGGGTCATAGTTCCCCTGAG |  |
| BG15671 | GGGCGTTGGTGATGTGGGCCCTCGTCATAGTTCCCCTGAG |  |
| BG15672 | CCACCTCCTCCTCCCGGTAAGGCGTCATAGTTCCCCTGAG |  |
| BG15674 | GTATGGCTTCATTCAGCTCCGGTTCGATTGGCTCCAATTCTTG |  |
| BG15675 | GAACCGGAGCTGAATGAAG |  |
| BG15677 | CCAACGATCAAGGCGAGTTAC |  |
| BG16494 | GTCTTCCACCCGGAGCACATCAAGGAGATCATGGCGGC |  |
| BG16495 | GAAAAGTTCTTCTCCTTTGCTCATGCCCCCAGGGTAGCATCATT |  |
| BG16496 | AATGATGCTACCCTGGGGGCATGAGCAAAGGAGAAGAACTTTTC |  |
| BG16497 | TGCGCCTGGGCATGGGTCAACTATATTTATTATTTGTAGAGCTCATCCATG |  |
| BG16498 | ATGGATGAGCTCTACAAATAATAAATATAGTTGACCCATGCCCAGG |  |
| BG16499 | GGGGAGGTCTTCAGGTCCACCACCATCCACGACCTGGAGCTTCTA |  |
| BG16500 | CGCCTTAGAAGCTCCAGGTCGTGGATGGTGGTGGACCTGAAGAC |  |
| BG16501 | TTTCGACGGAGGAGGCCTTGGCCGTCATAGTTCCCCTGAGATTATCGC |  |
| BG16504 | GCCGCCATGATCTCCTTGATGTGCTCCGGGTGGAAGAC |  |
| BG16505 | GGCCAAGGCCTCCTCCGTCGAAATCAACGCCCTTAACGGG |  |
| BG16545 | CCATGCAGTGGGACGACAC | Forward primer for colony PCR-based confirmation of the *TtarsM* deletion |
| BG16546 | TCACGGAGCAAGGAGAACT | Reverse primer for colony PCR-based confirmation of the *TtarsM* deletion |
| BG17206 | TGATCATGGACGTGACCACC | Forward primer for colony PCR-based confirmation of the *TtarsX* substitution by *syfp* |
| BG17207 | CCTTCTACATGGAGCCGGAC | Reverse primer for colony PCR-based confirmation of the *TtarsX* substitution by *syfp* |
| BG15624 | GGCCTGGACATCGTGAAGTTT | Forward primer for colony-PCR based confirmation of the curing of the pMK-ThermoCas9 vector |
| BG15625 | TCACAGGGGCCTGATCG | Reverse primer for colony-PCR based confirmation of the curing of the pMK-ThermoCas9 vector |
