# Supplementary material for: A Hyperthermoactive-Cas9 Editing Tool Reveals the Role of a Unique Arsenite Methyltransferase in the Arsenic Resistance System of Thermus thermophilus HB27
Source: mBio. 2021 Dec 7;12(6):e02813-21. doi: 10.1128/mBio.02813-21 (PMC8649762; doi:10.1128/mBio.02813-21)
Supplement: TABLE S4 [file mbio.02813-21-st004.docx]

**TABLE S4.**

| **Plasmid** | **Template** | **Primers** | **Product** |
| --- | --- | --- | --- |
| **ThermoCas9-targeting vectors** | | | |
| **pMK-ThermoCas9-NT** | *thermoCas9* fragment | BG15622 | *thermocas9* gene, codon harmonized for expression in *T. thermophilus* HB27 |
|  |  | BG15625 |  |
|  | pMK-Pnqo-syfp | BG15626 | pMK18 backbone (part 1) |
|  |  | BG15677 |  |
|  | *T. thermophilus* HB27 genome | BG15665 | 16S rRNA promoter |
|  |  | BG15669 |  |
|  | pThermoCas9_ctrl | BG15670 | Non-targeting spacer and sgRNA module |
|  |  | BG15674 |  |
|  | pMK-Pnqo-syfp | BG15675 | pMK18 backbone (part 2 and 3) and *nqo* promoter |
|  |  | BG15621 |  |
|  | | | |
| **pMK-ThermoCas9-sp1** | pMK-ThermoCas9-NT | BG15671 | Targeting spacer 1, sgRNA, pMK18 backbone (part 2 and 3) and *nqo* promoter |
|  |  | BG15621 |  |
|  | pMK-ThermoCas9-NT | BG15622 | *thermocas9* gene, pMK18 backbone (part 1) and 16S rRNA promoter |
|  |  | BG15666 |  |
|  | | | |
| **pMK-ThermoCas9-sp2** | pMK-ThermoCas9-NT | BG15672 | Targeting spacer 2, sgRNA, pMK18 backbone (part 2 and 3) and *nqo* promoter |
|  |  | BG15621 |  |
|  | pMK-ThermoCas9-NT | BG15622 | *thermocas9* gene, pMK18 backbone (part 1) and 16S rRNA promoter |
|  |  | BG15667 |  |
| **ThermoCas9-editing vectors** | | | |
| **pMK- ThermoCas9-HR-NT** | pMK-ThermoCas9-NT | BG15583 | pMK18 backbone (part 3), *nqo* promoter and *thermoCas9* gene |
|  |  | BG15625 |  |
|  | pMK-ThermoCas9-NT | BG15626 | pMK18 backbone (part 1), 16S rRNA promoter, spacer NT, sgRNA, pMK18 backbone (part 2) |
|  |  | BG15528 |  |
|  | *T. thermophilus* HB27 genome | BG15529 | *T. thermophilus* HB27 genomic region upstream the *TtarsM* gene |
|  |  | BG15580 |  |
|  | *T. thermophilus* HB27 genome | BG15581 | *T. thermophilus* HB27 genomic region downstream the *TtarsM* gene |
|  |  | BG15582 |  |
|  | | | |
| **pMK- ThermoCas9-HR-sp1** | pMK-ThermoCas9-sp1 | BG15583 | pMK18 backbone (part 3), *nqo* promoter and *thermoCas9* gene |
|  |  | BG15625 |  |
|  | pMK-ThermoCas9-sp1 | BG15626 | pMK18 backbone (part 1), 16S rRNA promoter, spacer 1, sgRNA, pMK18 backbone (part 2) |
|  |  | BG15528 |  |
|  | *T. thermophilus* HB27 genome | BG15529 | *T. thermophilus* HB27 genomic region upstream the *TtarsM* gene |
|  |  | BG15580 |  |
|  | *T. thermophilus* HB27 genome | BG15581 | *T. thermophilus* HB27 genomic region downstream the *TtarsM* gene |
|  |  | BG15582 |  |
|  | | | |
| **pMK-ThermoCas9-HR-sp2** | pMK-ThermoCas9-sp2 | BG15583 | pMK18 backbone (part 3), *nqo* promoter and *thermoCas9* gene |
|  |  | BG15625 |  |
|  | pMK-ThermoCas9-sp2 | BG15626 | pMK18 backbone (part 1), 16S rRNA promoter, spacer 2, sgRNA, pMK18 backbone (part 2) |
|  |  | BG15528 |  |
|  | *T. thermophilus* HB27 genome | BG15529 | *T. thermophilus* HB27 genomic region upstream the *TtarsM* gene |
|  |  | BG15580 |  |
|  | *T. thermophilus* HB27 genome | BG15581 | *T. thermophilus* HB27 genomic region downstream the *TtarsM* gene |
|  |  | BG15582 |  |
|  | | | |
| **pMK-ThermoCas9-HR-syfp** | pMK-ThermoCas9-NT | BG16500 | pMK18 backbone (part 3), *nqo* promoter, *thermoCas9* gene, pMK18 backbone (part 1), 16S rRNA promoter |
|  |  | BG16505 |  |
|  | pMK-ThermoCas9-NT | BG16501 | Spacer *yfp*, sgRNA, pMK18 backbone (part 2) |
|  |  | BG16504 |  |
|  | *T. thermophilus* HB27 genome | BG16494 | *T. thermophilus* HB27 genomic region upstream the *TtarsX* gene |
|  |  | BG16495 |  |
|  | pMK-Pnqo-syfp | BG16496 | *syfp* gene |
|  |  | BG16497 |  |
|  | *T. thermophilus* HB27 genome | BG16498 | *T. thermophilus* HB27 genomic region downstream the *TtarsX* gene |
|  |  | BG16499 |  |
