# Supplementary material for: A Hyperthermoactive-Cas9 Editing Tool Reveals the Role of a Unique Arsenite Methyltransferase in the Arsenic Resistance System of Thermus thermophilus HB27
Source: mBio. 2021 Dec 7;12(6):e02813-21. doi: 10.1128/mBio.02813-21 (PMC8649762; doi:10.1128/mBio.02813-21)
Supplement: TABLE S5 [file mbio.02813-21-st005.docx]

**TABLE S5.**

| ***thermoCas9*** |
| --- |
| atgaagtacaagatcggcctggacatcgggatcacctcgatcggctgggcggtgatcaacctcgacatcccgcggatcgaggacctgggcgtgcggatcttcgacagggccgagaaccccaagacgggcgagtcgcttgcgctgccgaggcggctggcccggtcggccaggaggaggctgcgcaggcggaagcacaggctggagcggatccggcggctgtttgtgcgggagggcatcctgaccaaggaggagctgaacaagctgttcgagaagaagcacgagatcgacgtgtggcagctgagggtcgaggcgctggacaggaagcttaacaacgacgagctggcccggatcctgctgcacctggcgaagcgcaggggcttcaggtcgaaccggaagtcggagcggacgaacaaggagaactcgaccatgctgaagcacatcgaggagaaccagtcgatcctgtcgtcgtacaggaccgtcgcggagatggtcgtgaaggaccccaagttctcgctgcacaagaggaacaaggaggacaactacacgaacacggtcgcccgggacgacctggagcgcgagatcaagctgatctttgccaagcagcgggagtacggcaacatcgtctgcacggaggcgttcgagcacgagtacatctcgatctgggcgtcccagcggccgttcgcgtcgaaggacgacatcgagaagaaggtgggcttttgcaccttcgagccgaaggagaagcgggccccgaaggcgacgtacacgtttcagtcgtttacggtgtgggagcacatcaacaagctgaggctggtgtcgcccggcgggatccgcgcgcttacggacgacgagaggaggctgatttacaagcaggcgttccacaagaacaagatcacgtttcacgacgtcaggacgctcctgaacctcccggacgacacgaggttcaagggcctgctgtacgacaggaacacgaccctgaaggagaacgagaaggtccggtttctggagctgggggcctaccacaagattcgcaaggccatcgacagcgtgtacgggaagggcgcggcgaagtcgttcaggcccatcgacttcgacacgttcgggtacgcgctgaccatgttcaaggacgacacggacatccggtcgtacctcaggaacgagtacgagcagaacggcaagaggatggagaaccttgccgacaaggtgtacgacgaggagctcatcgaggagctgctgaacctgtccttctcgaagttcggccacctttcgctgaaggccctgcggaacatcctgccgtacatggagcagggggaggtgtactcgacggcgtgcgagagggcgggctacacgttcacgggcccgaagaagaagcagaagaccgtcctcctgcccaacatcccccccatcgccaaccccgtggtgatgcgggcgctgacgcaggcgcggaaggtggtgaacgccatcatcaagaagtacgggtcgcccgtgtcgatccacatcgagctggcccgcgagctttcgcagtcgttcgacgagaggaggaagatgcagaaggagcaggagggcaacaggaagaagaacgagacggccatccggcagctggtcgagtacggcctgaccctgaacccgacgggcctggacatcgtgaagtttaagctttggagcgagcagaacggcaagtgcgcctactcgctgcagcccatcgagatcgagcgcctcctggagccggggtacacggaggtggaccacgtgatcccgtacagcaggagcctcgacgacagctacacgaacaaggtcctggtgctcacgaaggagaacagggagaagggcaaccggacgccggcggagtacctgggcctggggtcggagaggtggcagcagtttgagaccttcgtgctcacgaacaagcagttctccaagaagaagcgcgacaggctgctgcgcctgcactacgacgagaacgaggagaacgagttcaagaacaggaaccttaacgacacgaggtacatctcgcggtttctcgcgaacttcatccgggagcacctgaagtttgccgacagcgacgacaagcagaaggtctacaccgtgaacgggaggatcacggcccacctgcggagcaggtggaacttcaacaagaaccgcgaggagtccaacctccaccacgccgtggacgcggccatcgtggcctgcacgacccccagcgacatcgccagggtgacggccttttaccagcgccgggagcagaacaaggagctgtcgaagaagaccgacccccagttcccccagccgtggccccacttcgcggacgagctgcaggccaggctgtcgaagaacccgaaggagtcgattaaggcgctgaacctgggcaactacgacaacgagaagctggagtccctccagcccgtcttcgtgtcgaggatgcccaagcgcagcattacgggcgccgcgcaccaggagacgctccgcaggtacatcgggatcgacgagcgcagcggcaagattcagaccgtggtgaagaagaagctttcggagatccagctggacaagacgggccactttccgatgtacggcaaggagagcgacccgaggacgtacgaggccatccggcagcgcctcctggagcacaacaacgacccgaagaaggccttccaggagccgctgtacaagcccaagaagaacggcgagcttggcccgatcatcaggacgatcaagatcatcgacaccacgaaccaggtcatccccctgaacgacgggaagacggtggcctacaacagcaacatcgtgcgcgtggacgtgttcgagaaggacgggaagtactactgcgtgccgatctacacgattgacatgatgaagggcatcctcccgaacaaggccatcgagcccaacaagccctactcggagtggaaggagatgaccgaggactacacgtttaggttttcgctttacccgaacgacctgatcaggatcgagttccctagggagaagacgattaagacggcggtgggcgaggagatcaagatcaaggacctgtttgcctactaccagacgatcgactcgtcgaacggcggcctgtcgctcgtcagccacgacaacaacttctccctgcggagcatcggctcgaggacgctgaagaggtttgagaagtaccaggtcgacgtgcttgggaacatctacaaggtgaggggcgagaagagggtcggcgtggcctcgtcgtcgcactccaaggccggcgagacgatcaggcccctgtga |
